# A New Freshwater Biodiversity Indicator Based on Fish Community Assemblages

**DOI:** 10.1371/journal.pone.0080968

**Published:** 2013-11-22

**Authors:** Joanne Clavel, Nicolas Poulet, Emmanuelle Porcher, Simon Blanchet, Gaël Grenouillet, Sandrine Pavoine, Anne Biton, Nirmala Seon-Massin, Christine Argillier, Martin Daufresne, Pauline Teillac-Deschamps, Romain Julliard

**Affiliations:** 1 Department of Environmental Sciences, Policy and Management, University of California, Berkeley, Berkeley, California, United States of America; 2 Département Écologie et Gestion de la Biodiversité, Unité mixte de recherche 7204 : Université Paris 6 ; Centre National de la Recherche Scientifique, Muséum National d’Histoire Naturelle, Paris, France; 3 Département Ecohydraulique, Office National de l’Eau et des Milieux Aquatiques ; Institut des Mécaniques des Fluides ; Institut national de la Recherche en Sciences et Technologies pour l’Environnement et l’Agriculture, Toulouse, France; 4 Station d’Ecologie Expérimentale du CNRS, Unité de Recherche 2936 : Centre National de la Recherche Scientifique, Moulis, France; 5 Département Évolution et Diversité Biologique, Unité mixte de recherche 5174 : Université Paul Sabatier de Toulouse ; Centre National de la Recherche Scientifique, École Nationale de Formation Agronomique, Toulouse, France; 6 Department of Zoology, University of Oxford, Oxford, United Kingdom; 7 Department of Statistics, University of California, Berkeley, Berkeley, California, United States of America; 8 Département de l’Action Scientifique et Technique, Office National de l’Eau et des Milieux Aquatiques, Vincennes, France; 9 Département Eaux - Hydroécologie, Unité Recherche HYAX : Office National de l’Eau et des Milieux Aquatiques ; Institut national de la Recherche en Sciences et Technologies pour l’Environnement et l’Agriculture, Aix-en-Provence, France.; Consiglio Nazionale delle Ricerche (CNR), Italy

## Abstract

Biodiversity has reached a critical state. In this context, stakeholders need indicators that both provide a synthetic view of the state of biodiversity and can be used as communication tools. Using river fishes as model, we developed community indicators that aim at integrating various components of biodiversity including interactions between species and ultimately the processes influencing ecosystem functions. We developed indices at the species level based on (i) the concept of specialization directly linked to the niche theory and (ii) the concept of originality measuring the overall degree of differences between a species and all other species in the same clade. Five major types of originality indices, based on phylogeny, habitat-linked and diet-linked morphology, life history traits, and ecological niche were analyzed. In a second step, we tested the relationship between all biodiversity indices and land use as a proxy of human pressures. Fish communities showed no significant temporal trend for most of these indices, but both originality indices based on diet- and habitat- linked morphology showed a significant increase through time. From a spatial point of view, all indices clearly singled out Corsica Island as having higher average originality and specialization. Finally, we observed that the originality index based on niche traits might be used as an informative biodiversity indicator because we showed it is sensitive to different land use classes along a landscape artificialization gradient. Moreover, its response remained unchanged over two other land use classifications at the global scale and also at the regional scale.

## Introduction

In 2002, the 188 countries that are signatories to the Convention on Biological Diversity committed themselves to “achieve by 2010 a significant reduction of the current rate of biodiversity loss at the global, regional and national level” [1,2]. Even though this target was not achieved (the new target is 2020), research in the field of biodiversity indicators has been growing during the last decade [3,4]. 

As biodiversity is a complex object and subject, a first step for improving conservation plans is to build indices, which are intended to synthesize and simplify data in quantitative terms. Indices vary depending on the biological level quantified, i.e. from genes to biomes. Such a variety of biodiversity levels respond to the numerous ways of examining biodiversity, as defined by the Convention of Biological Diversity [5]. As indices quantify an aspect of biodiversity, they can become useful indicators if they tell us about the impact of human pressures on biodiversity. Facing global changes, species responses are not uniform [6]. Although a few species are not negatively affected by human activity and are flourishing, many are declining or will become extinct in the next century [7]. In this sense, the evaluation of biodiversity needs to move away from a reliance on species lists and case-by-case approaches to give a more global picture of what happens for most species in an ecosystem. 

Up until ten years ago, all river ecosystem indicators were assessed on their hydromorphological, chemical, and biological characteristics (e.g. *IBGN* [8] or *EPT* [9]). Because of their ability to integrate environmental variability at different spatial scales, fish assemblages have been studied and new indicators of river ecosystems have been developed (e.g. *IPR* [10], [11]). Although these indicators encompass the relative importance of geographic, ecoregional, and local factors, they were developed using the reference condition of pristine ecosystems without human impacts. As Baker and King (2013) point out, there is a crucial need for new aquatic indicators based on other criteria than biotic indices or summary metrics (e.g. taxon richness, ordination scores) especially in assessment and management [12]. Here, we develop a new approach, specifically dedicated to evaluate different functional approaches of fish biodiversity. It is the first study to synthesize comparisons between a large variety of fish traits: life history traits, morphological traits linked to habitat or diet, habitat niche, and an integrative approach based on abiotic habitat specialization. 

Community indices consider upper biological organization levels beyond the species level. They take into account the relationship between species inside the community, sometimes explicitly, such as in trophic networks [13,14], and sometimes implicitly, such as in niche or habitat specialization approaches [15]. Even if all community indices are species-based, they incorporate more complexity than species indicators because of these interactive approaches. They thus correspond more closely to a primary objective for indicators, producing a synthetic representation of biodiversity. Indeed, these indicators should help tackle the problem of maintaining the entire community integrity despite global changes by providing decision makers with more accurate information about human impacts on a global scale. In this respect, they are closer to the steady-state perspective, a frequently mentioned policy objective. It is easy to address the functional facet of biodiversity in this way and quite popular nowadays in the ecosystem “services” context [16]. However, basic summary metrics at the community level lose valuable information and non-linear declines should be undetected with aggregate responses [17]. In function of the study objectives it may be important, especially in conservation, to analyze the dataset species by species (or see TITAN, [17]) and it is always helpful to carefully interpret the community results. 

Finally, the criterion to create an indicator is to build good communication tools that are easy to understand and friendly to use, adapted to the context and scale of needs [18,19]. Indicators provide information to fuel dialogue between different scientific disciplines and stakeholders involved in biodiversity conservation. However, indicators also try to reach new targets identified as extremely important for the preservation of biodiversity. Indicators in this case attempt to open a dialogue and convince people not already involved in conservation including policy makers (local to international), judges, industrials, and farmers.

In this paper we aim to develop indices to better understand functional patterns in space and time of river fish communities and to evaluate their potential as biodiversity indicators for environmental policy makers. 

First, we quantified the spatial and temporal changes in composition of French fish communities with two different approaches: originality and specialization indices. For the originality indices, we used four sets of functional traits (habitat niche, life history, diet-linked morphology, and habitat-linked morphology) and the phylogeny to obtain five matrices for the twenty-six common fish species considered. We first used the metric of originality defined as the rarity of species traits to obtain scores for each species [20]. Thus, the whole contribution of species to trait community depends on its originality. More precisely, as integrative community-traits indices, we computed the average value of the originality score depending on the density of species locally present. The second approach was based on niche theory and species specialization such as it has been done for birds [21,22]. To carefully interpret the community results we also explored spatio-temporal analyses at the species level. We identified regions of low originality or specialization communities at the national scale and explored the temporal changes through nineteen years. For the first time, we explored potential congruent or mismatched patterns between different functional traits approaches. 

Next, we evaluated the link between these community indices and human pressures via land use. We used land use as our proxy because threats to global freshwater biodiversity are mainly due to industrial and agricultural impacts [23]. We tested the sensitivity of each of the six indices to human pressures using habitat modification data sets, and used these results to select biodiversity indicators. Finally, we discussed the choice of indicators selected by communication criteria to give a clear message for stakeholders and, especially in our case, for environmental policy makers.

## Materials and Methods

### Fish database

We worked with the database of the French National Agency for Water and Aquatic Environments (Onema), which contains records of standardized electrofishing protocols performed between 1990 and 2009 during low-flow periods (May-October). Electrofishing is considered the most effective non-destructive sampling procedure for describing fish assemblage structure [24]. Sampling protocols were defined depending on river width and depth. Streams were sampled by wading (mostly two-pass removal), while fractional sampling strategies were undertaken in larger rivers. Since the implementation of the EU Water Framework Directive’s surveillance monitoring, protocols follow the recommendations of the European Committee for Standardization [25]. To compare inter-annual densities, however, only surveys performed with the same sampling protocol were selected in the whole data set. Fishes were identified to species level, counted and then released. We worked with the 26 species for which trait data were available (around 90% of the total abundance catch) (Table 1). 

**Table 1:** List of the studied species.

**Latin Name**	**English common name**	**French common name**
*Abramis brama*	Common Bream	Brème commune
*Alburnoides bipunctatus*	Bleak	Spirlin
*Alburnus alburnus*	Bleak	Ablette
*Ameiurus melas*	Black bullhead	Poisson chat
*Anguilla Anguilla*	European Eel	Anguille
*Barbatula barbatula*	Stone loach	Loche franche
*Barbus barbus*	Common Barbel	Barbeau fluviatile
*Carassius spp.*	Crucian carp	Carassin
*Chondrostoma nasus*	Common nase	Hotu
*Cottus gobio*	European Bullhead	Chabot
*Cyprinus carpio*	Common carp	Carpe commune
*Esox lucius*	Northern Pike	Brochet
*Gasterosteus aculeatus*	Three spines stickleback	Epinoche
*Gobio gobio*	Gudgeon	Goujon
*Gymnocephalus cernuus*	Ruffe	Gremille
*Lepomis gibbosus*	Pumpkinseed sunfish	Perche soleil
*Leuciscus leuciscus*	Dace	Vandoise
*Perca fluviatilis*	European perch	Perche
*Phoxinus phoxinus*	Minnow	Vairon
*Pungitius pungitius*	Ninespine stickleback	Epinochette
*Rutilus rutilus*	Common roach	Gardon
*Salmo salar*	Atlantic salmon	Saumon atlantique
*Scardinius erythrophthalmus*	Common rudd	Rotengle
*Squalius cephalus*	European chub	Chevaine
*Telestes soufia*	Souffia	Blageon
*Tinca tinca*	Tench	Tanche

We extracted collecting events from Onema’s fish database using two different criteria:

(i) All sites regardless of temporal coverage, which yielded 5 403 sites with 1–20 years of sampling and a total of 13 076 sampling occasions (Dataset 1).
(ii) Only sites with at least 8 years of data, which yielded 557 sites with 8–20 years of sampling from 1990 to 2009 and a total of 6942 sampling occasions (Dataset 2; see [26]).

### Trait dataset

#### (i): Habitat use

This dataset consists of five parameters describing the habitat use of a river species: foraging habitat, reproductive habitat, position in the water column, salinity tolerance and rheophily (the ability to live in fast moving water). The information has been gathered from different sources [27,28].

#### (ii): Life history traits (LHT)

The life history traits included in the study were: maximum lifespan, female age at maturity, number of spawns per year, logarithm of the maximum body length, relative fecundity (Number of ovocytes per gram), egg diameter, and parental care [27-29].

#### (iii): Morphological database

**Figure 1:**
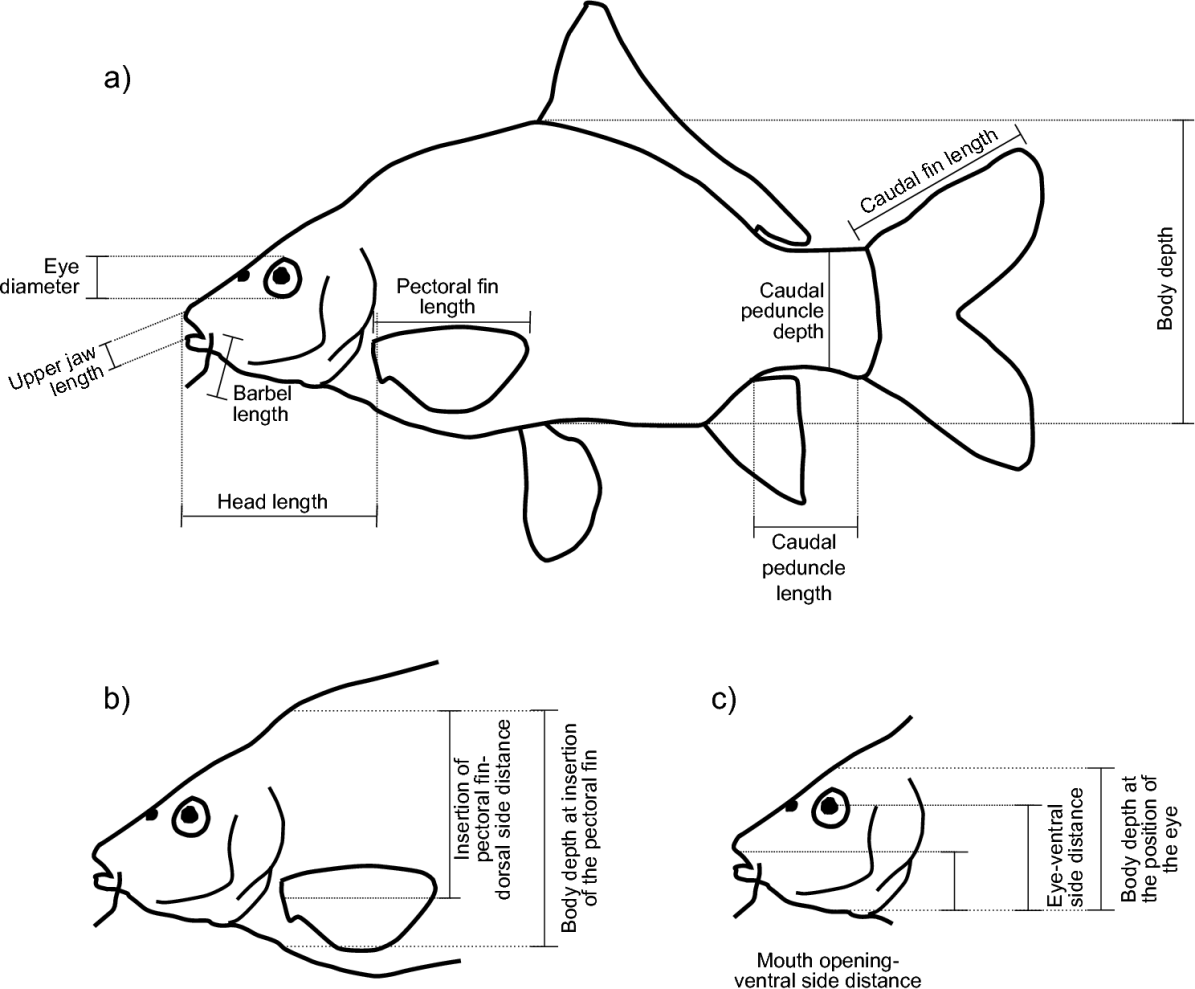
Functional character measurements. a) All measurements are standardised by the standard length. Caudal peduncle length is also standardised by body depth; caudal peduncle depth was only standardised by body depth; b) pectoral fin position: pectoral fin dorsal side distance divided by body depth at pectoral fin; c) eye position: eye–ventral side distance divided by body depth at the eye; mouth position: mouth–ventral side distance at the position of the eye divided by body depth at the position of the eye.

Fourteen morphological traits related to two different axes of the niche (diet and habitat) were used (Figure 1, Table 2) [30-32]. Traits were measured from pictures collected mainly from FishBase [33,34], but for details see 35. All traits were standardized to account for differently sized photographs and species (e.g. standard length). 

**Table 2:** Description of functional traits related to the habitat and diet niche axes [30-32]. From Schleuter et al. [35].

**Niche axis**	**Morphological Traits**	**Code**	**Functional Interpretation**
	Pectoral fin length	PL_SL	Maneuvering speed, habitat velocity
**Habitat**	Vertical position of pectoral fin	PFP	Turning capacity
**and**	Body Depth	BD	Maneuvering, hydrodynamics in the habitat
**Swimming**	Caudal peduncle Length / BD	CpD	Swimming ability
**ability**	Caudal peduncle Depth / BD	CpL	Swimming ability
	Caudal peduncle length	CL	Swimming speed
	Eyes Position	EP	Vertical position in the Water column
	Eyes diameter	ED_SL	Adaptation (i) light (turbidity and diurnal) (ii) Relative prey size
**Diet**	Mouth Position	MP	Location of food acquisition
**and**	Length of longest barbell	BarL_SL	Non visual food detection, benthic feeders
**Food**	Head length	HL_SL	Relative prey size
**acquisition**	Length of upper jaw	MS	Relative prey size
	Maximum size	Lmax	Actual prey size (in combination with head and upper jaw length)

From Schleuter et al. [35].

#### (iv): Phylogenetic dataset

We retrieved molecular data from three mitochondrial genes from GenBank (cytochrome b, cytochrome oxidase I and ribosomial 16S sub-unit). We inferred the best evolutionary model for each gene using maximum likelihood methods implemented in Paup4 ob10 [36]. The best model of molecular evolution was obtained using Modeltest based on the AIC criterion [37], (for more details see 38). 

### Human Pressure dataset

The dataset of human pressures was provided by the European land-cover database CORINE, which classifies landscape units larger than 25 ha into one of 44 classes [39] on the basis of satellite digital images (e.g. SPOT and LANDSAT). We used the 2000 update and considered three alternative groupings of seven habitat classes: (i) The CORINE Land Cover (CLC) yields 5 habitat classes: “Forest”, “Meadow”, “Farming”, “Urban”, and a “Mix” (i.e. a Mix between agricultural and urban habitats) (ii) The EUROWATER (a special variant of CLC for freshwater common to the European scale), yields 6 habitat classes with the addition of the “Intensive Urban” habitat class, and (iii) The ONEMA land use classification (a special variant of CLC and EUROWATER for freshwater common to the national scale) yields 7 habitat classes with the addition of the “Intensive Farming” class. Only the latter two classifications are used to test the reproducibility of our indicator. 

Here, we consider land use classification as a gradient of landscape artificialization under human pressures. Land use is a common proxy for human pressures in terrestrial communities [6,22]. The link between land use and human pressures in river has been reviewed at a global scale [40], but also on regional scale in North America [41,42] and Europe [43]. Marzin and al. (2013) showed a clear link between the CORINE Land Cover (CLC) dataset and different pollution and physical modifications at both local and regional scales [43]. If both human pressures are correlated with CLC, water quality parameters are more strongly correlated to land use than physical modifications.

### Statistical Analyses

All statistical analyses were performed using R 2.15.1 (R Development Core Team. 2012), and more particularly the ade4 and nlme packages [44,45]. We calculated one index for each kind of data set, thus for a total of 6 indices: 4 functional originality indices, 1 phylogenetic originality index, and 1 specialization index.

#### (i): Functional and phylogenetic originality

To characterize the functional originality of each species, we used the mean of a set of functional traits from the different datasets described above. For each dataset a distance matrix was created using the Gower's dissimilarity index to allow the treatment of various statistical types of variables when calculating distances [46]. A hierarchical clustering (the unweighted pair-group clustering method using arithmetic averages: UPGMA) of the distance matrix produced a functional dendrogram comprising all the species. For each functional tree and the phylogenetic tree we used the procedure of Pavoine et al. [20] to estimate the biological originality of each species using the quadratic entropy of Rao [47]. Branch lengths and tree topology are jointly taken into account in the calculation of this index of originality. We computed both the Equal-split index [48] and the QE-based index [20]. The Equal-split index is more influenced by unique traits (trait states observed in a single species) than rare traits (trait states shared by a few species), whereas the reverse is true for the QE-based index. However, as both indices yielded similar results, we retained the equal-split index only, which is subsequently referred to as Species Originality Index (SOI). When it was possible we explored the sensitivity of our SOI to the addition of species in the data set [see the File S1].

#### (ii): Species Specialization Index

Ideally, specialization should be measured as the multi-dimensional breadth of a species’ ecological niche. An integrative index of habitat specialization (Species Specialization Index, SSI) was developed for birds [21], as the coefficient of variation (standard deviation/average) in average density of a species across habitats. We tested the relevance of this index in fishes. Because ecological habitat classes were missing for several species, we used habitat traits and four abiotic variables: temperature (sum of January to June air temperatures), longitudinal gradient, log of elevation, and slope (see 13 for more details). We had to take into account the geographical bias in the data set. This bias was linked to an over-sampling of headwaters. We therefore reassigned all the sampled points into 7 habitat classes with an approximately equal amount of samples in each habitat class.

#### (iii): Community Indices

Each species can be ranked along a continuous gradient from the least to the most original or specialized species (X_1_,…, X_i_). Any species assemblage at time *t* can be characterized by the average specialization or originality taken across all individuals in the assemblage. These community level indices are simple weighted averages, i.e. ∑(*a*_*i*,*t*_ *X*_*i*_)/∑*a*_*i*,*t*_, where *a*_*i*,*t*_ is the relative abundance of species *i* in the assemblage at time *t* and *X*_*i*_ the originality or specialization of species *i*. In the following, CSI_*t*_ = ∑(*a*_*i*,*t*_ *SSI*_*i*_)/∑*a*_*i*,*t*_ is the Community Specialization Index and COI = ∑(*a*_*i*,*t*_ *SOI*_*i*_)/∑*a*_*i*,*t*_, the Community Originality Index at time *t*. We explored the temporal and spatial variation of both community indices, COI and CSI, using mixed-effects linear models with sampling site as a random effect [49,50] and Akaike Information Criteria (AIC) model selection. We also explored the link of all the community indices between them by exploring their correlations by performing linear model. For the statistical independence of the data spatio-temporal effects and their interactions were taken into account, and we selected the model in function of its AIC. 

#### (iv): Community Indicators

**Figure 2:**
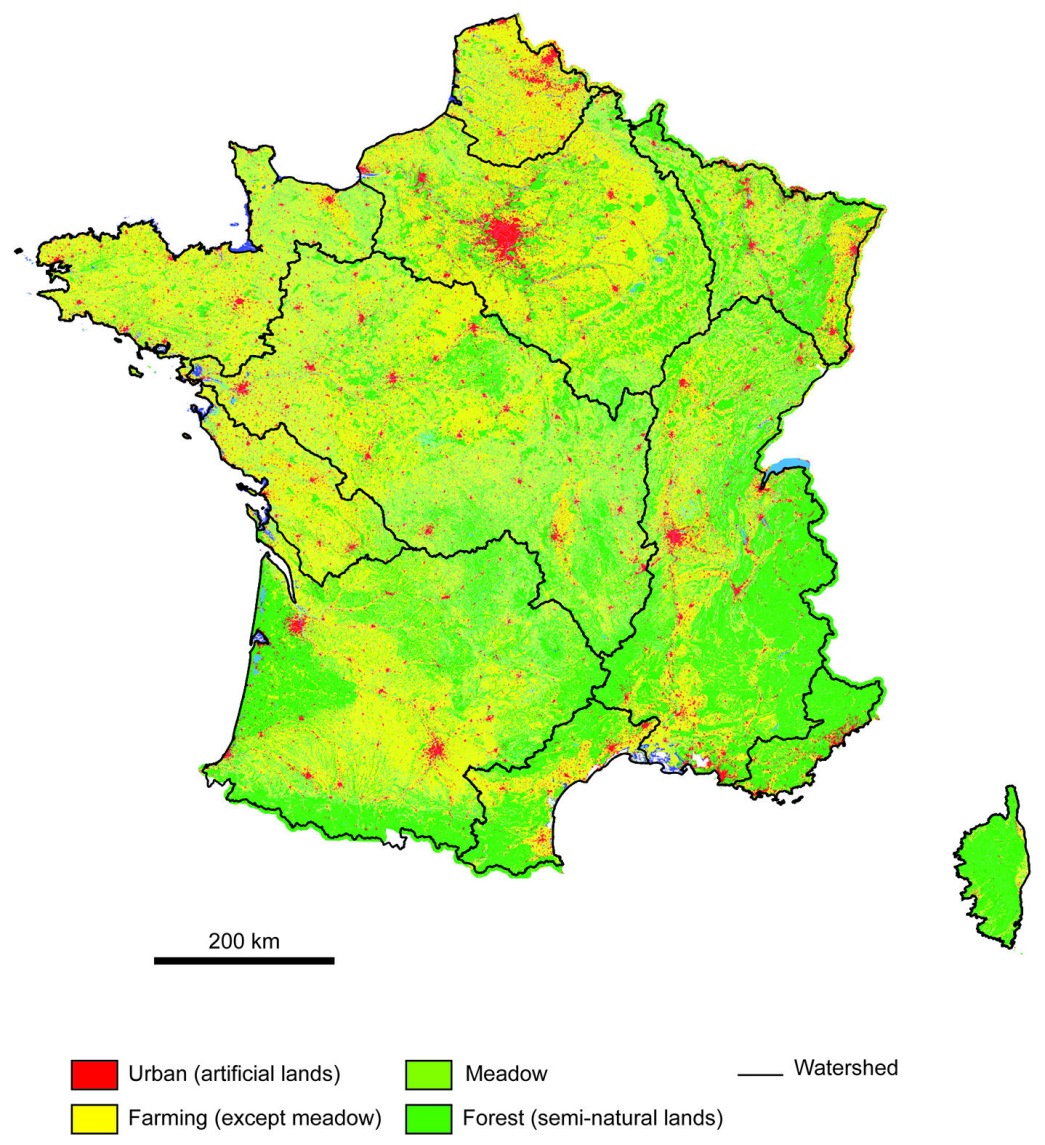
Spatial distribution of human pressures. We used the CORINE Land Cover data set and its land use classification as an artificialization gradient from natural habitat to urban habitat. Colors from green to red represent increasing pressures.

We tested the relationships between CSI and five COI (four functional and one phylogenetic originality indicators) and landscape variables using mixed-effects linear models with sampling site as a random effect. Temporal (year) and spatial effect (geographical coordinates and watersheds) with their interactions were also taken into account. Because no R-squared can be calculated with random effect, we only obtained a proxy of the R-squared with the same model without the random effect. We used the CORINE Land Cover dataset (Figure 2) and its two variations to evaluate the reproducibility of our results and thus the sensitivity of each community index through habitat classifications. Then we studied the scale dependence of the community index response by exploring the relationship at the regional watershed scale.

## Results

### (i). Species Originality and Specialization Indices

**Figure 3:**
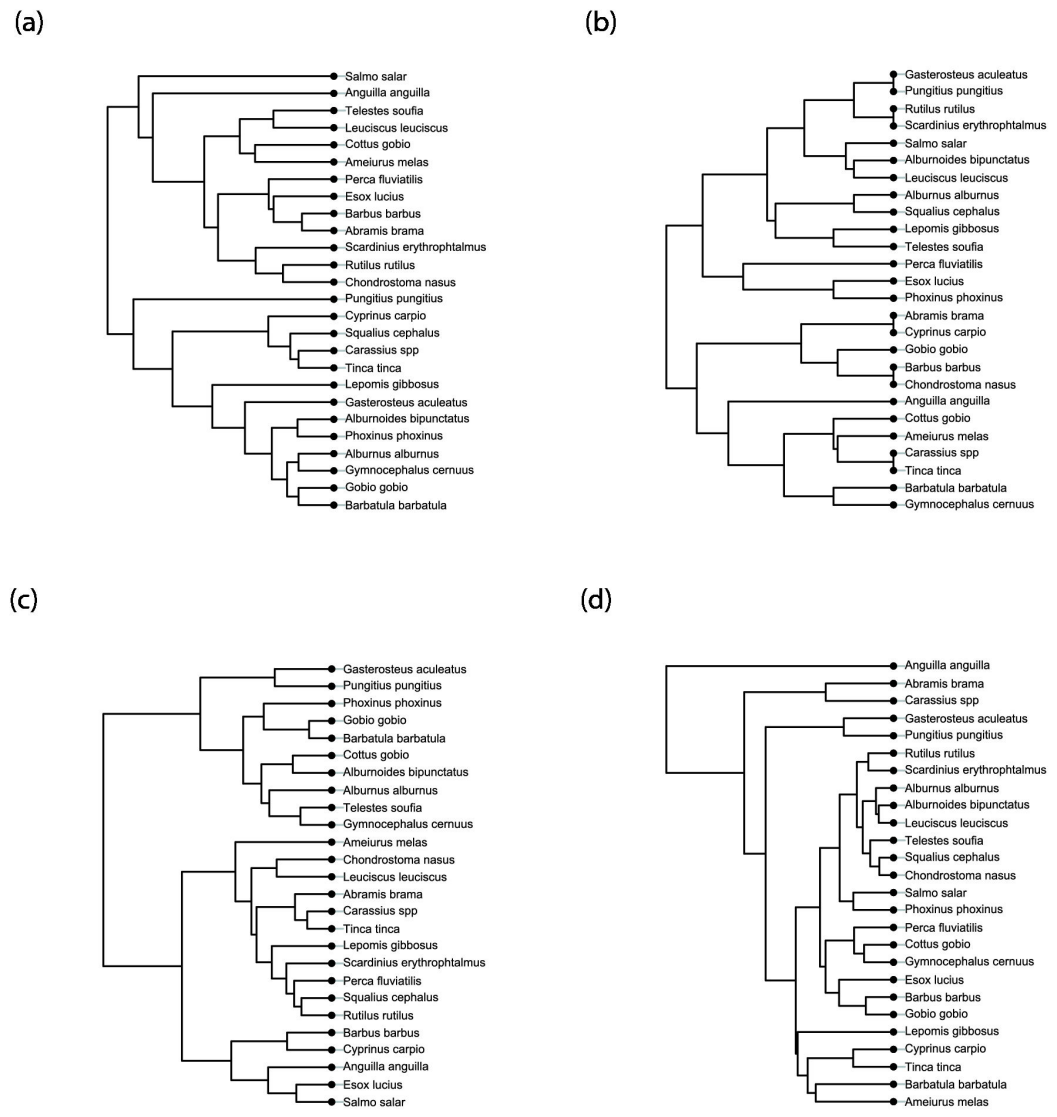
Functional trees. (a) Tree based on Life History Traits (b) Tree based on functional Niche traits (c) Tree based on functional Diet Morphological traits (d) Tree based on functional habitat-linked morphological traits.

**Figure 4:**
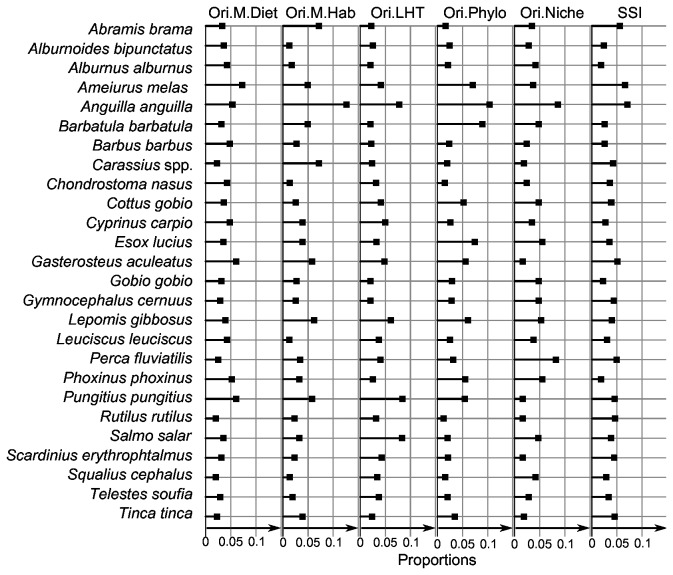
Results of species values of originality and specialization in a Cleveland’s dot plots in proportions (i.e. the sum of species values is one for each index).

The four trait distance matrices can be visualized using trees (Figure 3). Trees based on life-history traits (Figure 3a), functional niche (Figure 3b), and diet-linked morphological traits (Figure 3c) were well balanced in the sense of Blum et al. [51]. These authors defined the balance of a tree as the average balance of its nodes, “assuming that a given node is completely balanced if it splits the sample into two subsamples of equal size”. At the opposite, the tree based on habitat-linked morphological traits (Figure 3d) was highly unbalanced by the European eel (*Anguilla anguilla*), and, to a lesser extent by the groups common bream (*Abramis brama*), crucian carp (*Carassius* sp.), ninespine stickleback (*Pungitus pungitius*), three spines stickleback (*Gasterosteus aculeatus*). Using the equal-split metric, we computed four originality indices for each species to evaluate the three functional datasets and the phylogeny (Figure 4). As expected, *A. anguilla* was characterized by a high originality score for the habitat-linked morphological trait matrix (SOI = 0.81 compared to a mean of 0.24). The two other imbalanced nodes had smaller originalities (*Abramis* group = 0.46 and *Pungitius* group = 0. 37). The species specialization index ranked the European eel as the most specialist species and the common bleak, *Alburnus alburnus* as the most generalist species (Figure 4). At the community level, the habitat-linked morpho-COI was sensitive to the presence of the European eel and to a lesser extent to the presence of the common bream, *Abramis brama*, and the crucian carp, *Carassius* sp. We tested the sensitivity of the Species Originality Index (SOI) based on traits to the addition of species in the initial dataset. The life history traits index and the habitat-linked morphological index were strongly correlated (respectively R^2^=81, R^2^=86). The diet-linked morphological index and the niche index were less correlated (respectively R^2^ = 65 and R^2^=68) and thus more sensitive to the addition of fish species in the initial dataset [see the File S1].

### (ii). Community Indices: spatial and temporal patterns

**Figure 5:**
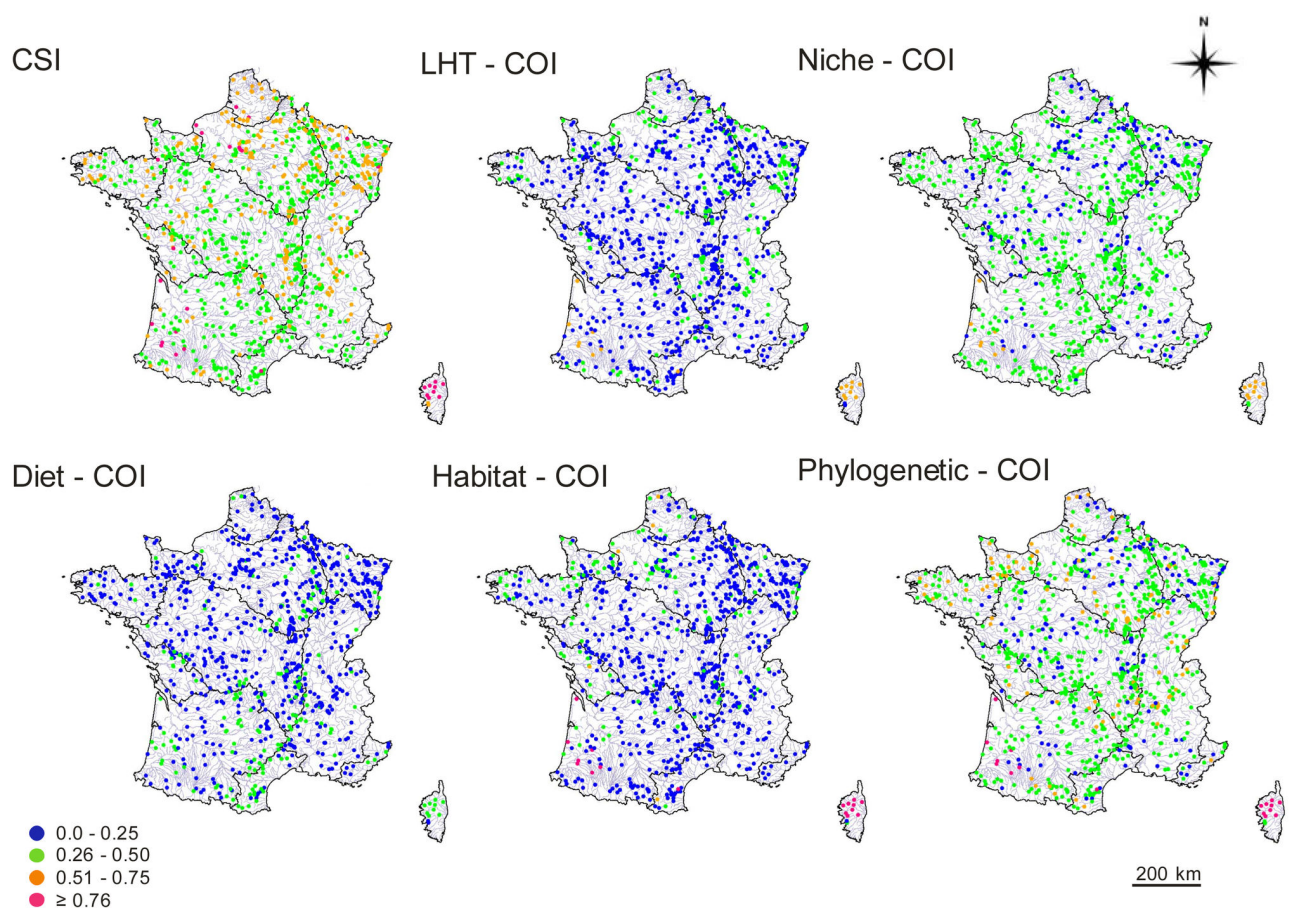
Spatial distribution of community specialization (CSI) and originality (COI) indices for 2005 (the years with the most sample stations). Colors from green to red represent increasing index values.

All statistical models retained by the AIC, with both datasets, contained the same variables: geographic coordinates, year, and their interaction, except for the Diet-COI where the watershed effect gave a better model (AIC). Because Corsica appeared to be an outlier (Figure 5), we re-ran all analyses excluding data from this area. With Corsica excluded, we found that watershed was a better spatial effect than geographic coordinates (AIC). 

Corsica clearly comes out as a hotspot of fish originality and specialization (Figure 5). In contrast, the Seine watershed presented the lowest originality and the most generalist fish communities. The CSI, habitat-linked morpho–COI and LHT–COI presented limited variation among sites and appeared to be ineffective to discriminate sites. In contrast, the diet-linked morpho -COI highlighted a strong originality in all small rivers, especially mountainous streams. Although the temporal effect was always retained in statistical models on the AIC, it was not always significant. However, Niche and both Morpho-COI increased significantly over the last years (Table 3).

**Table 3:** Results on temporal effect for three functional community indices, all the other indices present no temporal effect.

**Temporal main effect**		**Dataset1**	**Dataset2**
***COI- Niche***	Coef.	0.002	0.002
Year*(x + y)	t value	2.65	2.32
	p value	0.008	0.02
***COI- Mhab***	Coef.	0.004	0.005
Year*(x + y)	t value	3.67	3.11
	p value	<0.001	0.002
***COI- Mdiet***	Coef.	0.005	0.003
Year*(x + y + w)	t value	3.45	2,09
	p value	<0.001	0.04

The main temporal effect is given for each best model selected on the AIC, each statistical model has been run with a station as a random effect and as a fixed effect time and space and their interactions. The main difference is from the final space effect, which could be abscissa (x), ordinate (y), watershed (w) effect and all possible combinations. Results are similar without Corsica Island.

### (iv). Community Indicators

Our results showed that all COI, which are based on the same originality metrics, tended to be correlated with each other, while the CSI, based on a different approach and metric, was correlated with a subset of the COI only. All these correlations took into account space and time effect for the statistical independence of data. The habitat-linked morpho –COI was correlated with the LHT – COI (R^2^= 0.47, F_8,12829_ = 1407, P < 0.001), and with the diet-linked morpho –COI (R^2^= 0.24, F_8,12829_ = 518, P < 0.001). Interestingly, the phylogeny approach seemed to capture different proportion of morphological variation in function of the diet or the habitat niche axis [phylo –COI and habitat-linked morpho -COI (R^2^= 0.53, F_8,12829_ = 1842, P < 0.001), and phylo-COI and diet-linked morpho COI (R^2^= 0.39, F_8,12829_ = 1037, P < 0.001)]. Unlike other vertebrates such as birds or mammals (birds: [52]), the fish Life History Traits index presented a weak correlation with the phylo-COI (R^2^= 0.13, F_8,12829_ = 239, P < 0.001). The niche-COI was more strongly correlated with the phylogenetic index (R^2^= 0.56, F_8,12829_ = 2013 10^4^, P < 0.001) than with the morphological indices (Diet: R^2^= 0.33, F_8,12829_ = 798, P < 0.001; and Habitat: R^2^= 0.50, F_8,12829_ = 1596, P < 0.001) even though the latter are assumed to represent niche axes. The niche-COI was also correlated with the LHT –COI (R^2^= 0.23, F_8,12829_ = 475, P < 0.001). 

The CSI was strongly correlated with the LHT-COI (R^2^= 0.74, F_8,12829_ = 3.43 10^4^, P < 0.001) and habitat-linked morpho - COI (R^2^= 0.47, F_8,12829_ 1397, P < 0.001) but weakly with the niche (R^2^= 0.11, F_8,12829_ 208, P < 0.001), and not with the diet-linked morpho (R^2^= 0.04, F_8,12829_ 65, P < 0.001) and phylo – COI (R^2^= 0.08, F_8,12829_ 149, P < 0.001). It is important to note that the level of specialization measured here is more relevant to describe the Fish Life History Traits component than the habitat niche component.

**Figure 6:**
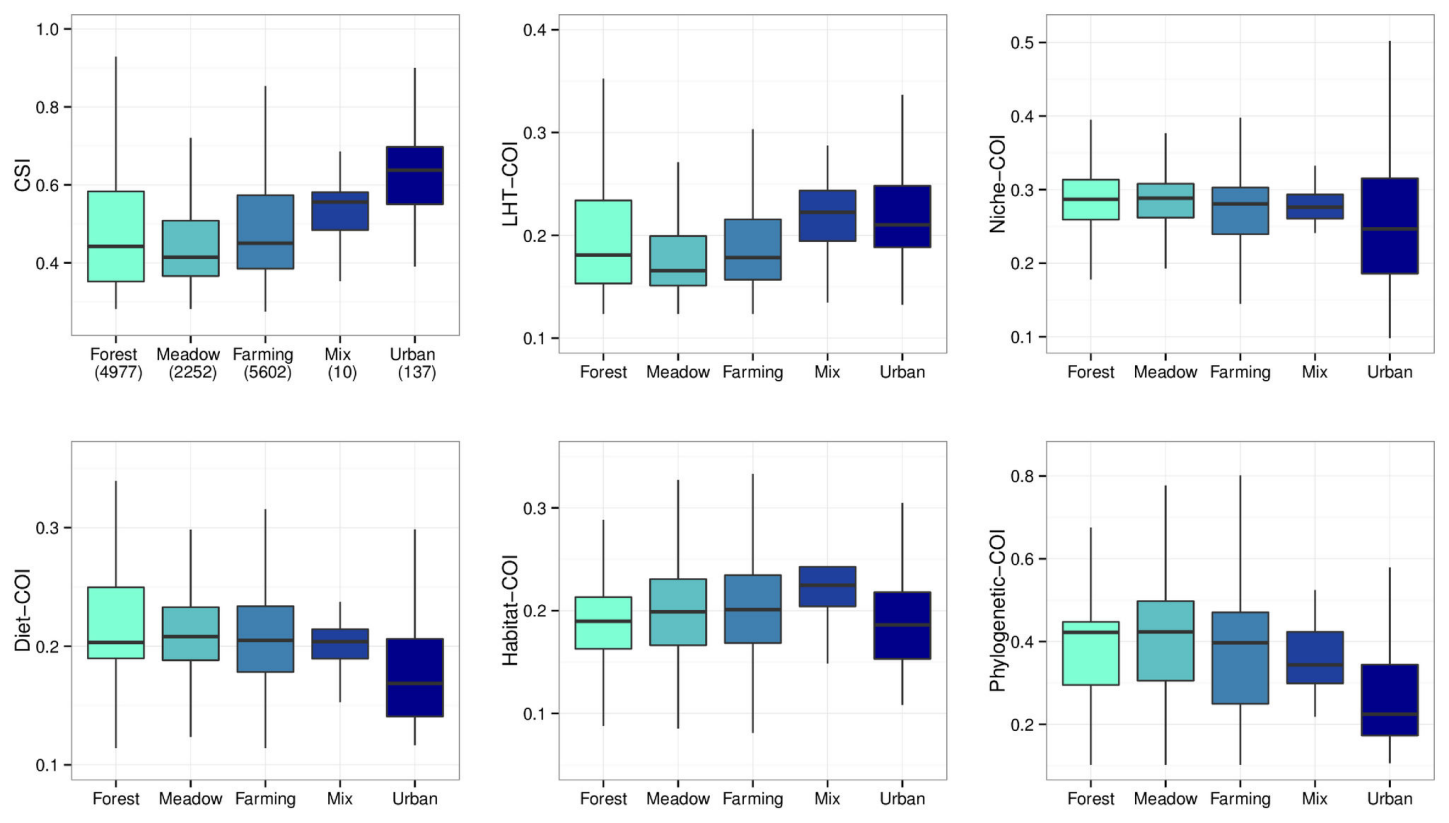
Community indices response to land use artificialization with the CORINE Land Cover dataset. Colors from green to blue represent increasing pressures.

**Figure 7:**
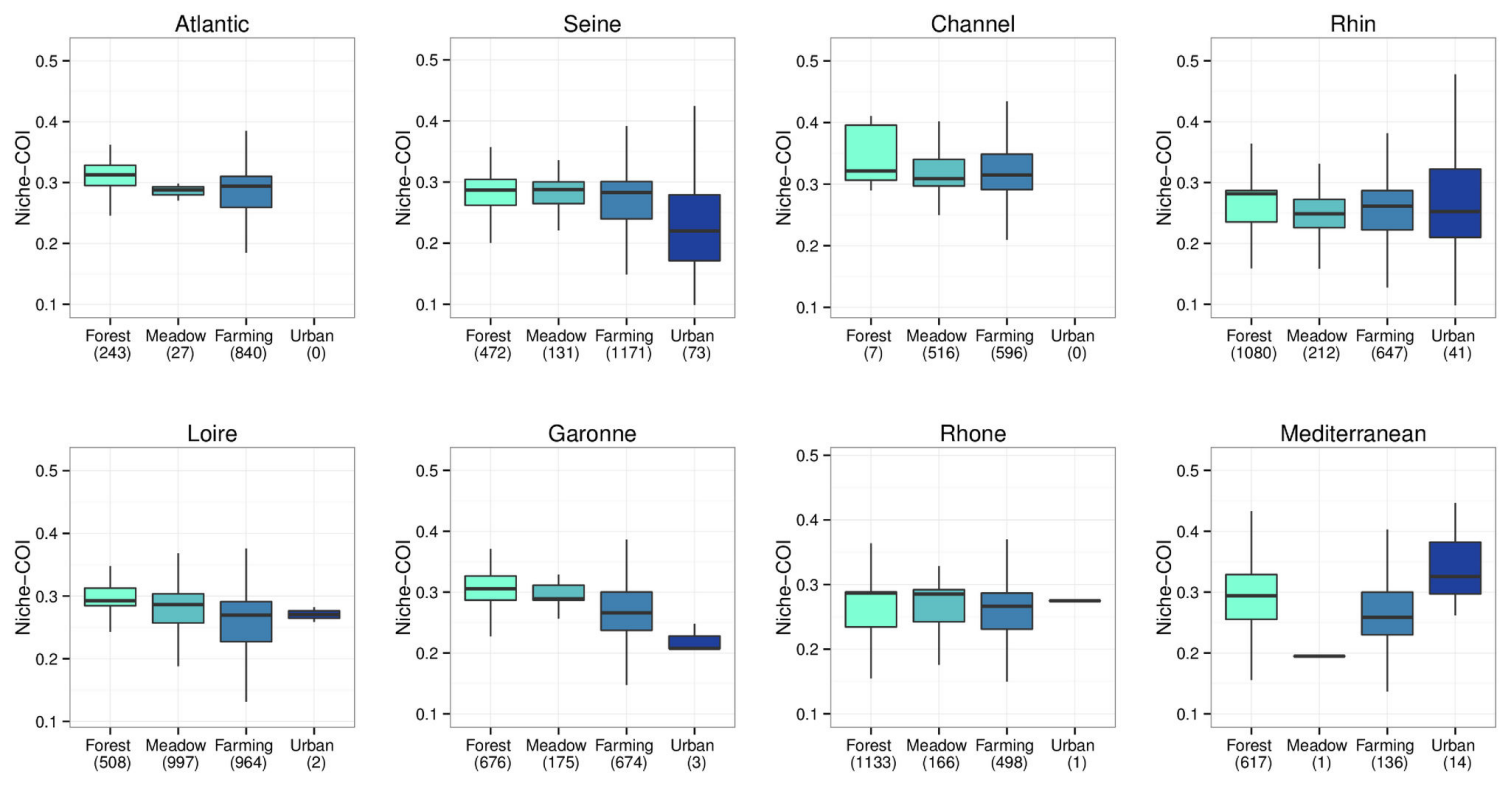
Niche-COI response to land use artificialization across watersheds. The Mix class is not represented because of limited sample size.

The sensitivity to human pressures of the six community indices was evaluated with respect to land use data and two variations of CORINE Land Cover (Table 4). All indices correlated with land use (Table 4-6), but with some variation. For example, some indices were sensitive to the different human pressures (farming or urban) represented here by an artificialization gradient (Figure 6). In contrast, the CSI was significantly higher for urban area than for agricultural or natural habitats (Figure 6). We used two variations of CORINE Land Cover (ONEMA and EUROWATER) to get an estimate of the community indices reproducibility in function of the arbitrary habitat classifications [Table 4-6, see the File S2] and only one COI was robust to the effect of habitat classifications: the Niche – COI (Table 4-6). The response of this latter index was also significantly sensitive to the different type of human pressures with a consistent behavior at national and regional scales (Figure 7). Within each watershed or over all watersheds the relationship between human pressures and Niche-COI is negative when it is expected to be negative (e.g. under human pressures like farmland and urban habitat) and reciprocally (e.g. under natural habitat).

**Table 4:** Sensitivity of community indices to human pressures as defined and measured in CORINE Land Cover.

**Index**	**Value**	**Meadow**	**Farming**	**Mix**	**Urban**
CSI	Coef	-0.04	NS	NS	0.1
R^2^ = 0.26	t-value	-7	NS	NS	8
	p-value	<0.001	NS	NS	<0.001
LHT-COI	Coef	-0.03	-0.01	NS	0.03
R^2^ = 0.28	t-value	-10	-7	NS	4
	p-value	<0.001	<0.001	NS	<0.001
Niche-COI	Coef	-0.01	-0.02	NS	-0.02
R^2^ = 0.27	t-value	-4	-10	NS	-3
	p-value	<0.001	<0.001	NS	0.04
Diet-COI	Coef	-0.006	-0.008	NS	-0.03
R^2^ = 0.06	t-value	-3	-6	NS	-6
	p-value	0.006	<0.001	NS	<0.001
Habitat-COI	Coef	NS	NS	NS	0.06
R^2^ = 0.38	t-value	NS	NS	NS	4
	p-value	NS	NS	NS	<0.001
Phylogenetic-COI	Coef	NS	-0.03	NS	-0.1
R^2^ = 0.21	t-value	NS	-6	NS	-5
	p-value	NS	<0.001	NS	<0.001

The relation between the community indices and the land used modification has been performed with a mixed-effects linear model with sampling site as a random effect and temporal (year) and spatial effect (geographical coordinates or watershed) and their interactions. The land use effects are given for each best model selected on the AIC. The coefficient effect of each habitat class is relative to the natural class (thus forest coefficient is always 0). We corrected the p-values for multiple tests using the Benjamini Yekutieli False Discovery Rate. We added the Rsquared adjusted from the equivalent linear model.

**Table 5:** Sensitivity of community indices to human pressures as defined and measured in EUROWATER, a special variant of CLC for freshwater common to the European scale.

**Index**	**Value**	**Meadow**	**Farming**	**Mix**	**Urban**	**Intens. Urban**
CSI	Coef	NS	NS	0.09	0.04	0.09
R^2^ = 0.27	t-value	NS	NS	10	5	9
	p-value	NS	NS	<0.001	<0.001	<0.001
LHT-COI	Coef	-0.008	-0.01	0.02	NS	0.01
R^2^ = 0.27	t-value	-3	-4	4	NS	3
	p-value	0.003	0.002	<0.001	NS	0.01
Niche-COI	Coef	-0.01	-0.02	-0.01	-0.007	-0.01
R^2^ = 0.27	t-value	-5	-7	-4	-2	-3
	p-value	<0.001	<0.001	0.003	0.03	0.008
Diet-COI	Coef	-0.01	-0.009	-0.01	-0.02	-0.02
R^2^ = 0.06	t-value	-7	-4	-4	-9	-6
	p-value	<0.001	<0.001	<0.001	<0.001	<0.001
Habitat-COI	Coef	NS	-0.01	0.05	0.03	0.05
R^2^ = 0.39	t-value	NS	-3	8	5	7
	p-value	NS	0.02	<0.001	<0.001	<0.001
Phylogenetic-COI	Coef	-0.03	-0.03	NS	-0.02	-0.03
R^2^ = 0.20	t-value	-4	-4	NS	-3	-3
	p-value	<0.001	<0.001	NS	0.009	0.02

(See table 4 for details.)

**Table 6:** Sensitivity of community indices to human pressures as defined and measured in ONEMA, a special variant of CLC and EUROWATER for freshwater common to the French national scale.

**Index**	**Value**	**Meadow**	**Farming**	**Intens. Farming**	**Mix**	**Urban**	**Intens. Urban**
CSI	Coef	-0.03	NS	NS	0.08	0.02	0.07
R^2^ = 0.27	t-value	-5	NS	NS	9	3	8
	p-value	<0.001	NS	NS	<0.001	0.004	<0.001
LHT-COI	Coef	-0.03	-0.02	-0.02	0.01	NS	NS
R^2^ = 0.28	t-value	-10	-6	-6	3	NS	NS
	p-value	<0.001	<0.001	<0.001	0.02	NS	NS
Niche-COI	Coef	-0.01	-0.02	-0.02	-0.01	NS	-0.01
R^2^ = 0.28	t-value	-4	-9	-9	-4	NS	-3
	p-value	0.002	<0.001	<0.001	0.001	NS	0.01
Diet-COI	Coef	-0.008	-0.01	-0.007	-0.008	-0.02	-0.01
R^2^ = 0.06	t-value	-4	-6	-3	-3	-8	-5
	p-value	<0.001	<0.001	<0.001	0.006	<0.001	<0.001
Habitat-COI	Coef	NS	NS	-0.01	0.05	0.03	0.05
R^2^ = 0.39	t-value	NS	NS	-3	8	6	8
	p-value	NS	NS	0.02	<0.001	<0.001	<0.001
Phylogenetic-COI	Coef	NS	-0.04	-0.03	NS	-0.02	-0.02
R^2^ = 0.20	t-value	NS	-6	-4	NS	-2	-2
	p-value	NS	<0.001	<0.001	NS	NS	0.02

(See table 4 for details.)

## Discussion

The aim of this study was to develop new functional indices for river fish and to evaluate their potential application as functional biodiversity indicators. For the first time in fish communities, we examine spatio-temporal patterns of six functional facets of biodiversity relying on two different theoretical approaches: specialization and originality. We identified common conservation priorities but also spatial mismatching in function of the trait considered. Then, we linked them with human land use pressures and we identified the community functional originality index based on niche traits as the most likely to become a functional biodiversity indicator. Its sensitivity to the nature and intensity of human disturbance, considered here by an artificialization gradient, at both regional and national scales, results in a simple message to communicate with policy makers and biodiversity managers.

### I. Community Indices

There is a growing consensus that functional diversity based on species traits is a better predictor of ecosystem functioning than species number per se [53]. Species richness is currently the most used biodiversity index (and indicator) but it is highly scale-dependent, with local increases that are often accompanied by regional or global decreases and increases in between-site similarity [54]. Particularly, fish species richness tends to increase from upstream to downstream [55]; and the upstream part of many French rivers sustain only a few fish species (< 5 fish species, [56]). Species richness is thus an inadequate surrogate in the context of ecosystem function unlike community traits approaches, which appear more and more relevant in the literature especially to examine ecosystem integrity [16,22,57]. Community-trait indices take into account the species present in the area considered, species being grouped depending on their ecological or phylogenetic affinity. These indices compute the average value for a trait or character, depending on the frequency of species locally present. So, we did not consider intra-specific variability, which sometimes represents a significant proportion of the variability and complex spatio-temporal dynamic [58,59]. Even though specialization community indices seem to give the same message with presence/absence data [60], it is not always the case when a process or a function is measured using functional traits [19]. Moreover one of the most interesting points to use common species is based on the assumption that abundance plays an important role in ecosystem functioning [57,61].

Community Specialization Index (CSI) is a different approach than Community Originality Index (COI) approaches. If CSI is not clear on the underlying mechanisms explaining the precise ecosystem function, its well-known power comes from its holistic habitat approach. The central focus of CSI is not the species feature but its interaction with the environment by the habitat approach. This statement is closer to the Grinnell niche theory approach than the Hutchinson one [62]. And thus, in the CSI approach, the crucial point is the relevance of habitat description, not the species traits data set. 

On the other side, with the COI we study the distinctiveness of precise species traits and lineages, and thus we postulated that trait variation among species variation relates to functional differences in the ecosystem, which allows an interpretation in terms of ecosystem function or “services”. The set of traits selected is a crucial step toward this goal, especially if we want the dynamics of the indices to reflect ecosystem function [57]. Here, we worked only with traits having a demonstrated functional role in fish biology. For example, morphological traits such as the mouth position or the length of barbell are linked to the diet and food acquisition [29,35]. Because our originality index is based on distance metrics we verified that it was not species richness dependant of the initial dataset. The life history traits index and the habitat-linked morphological index are less sensitive (R^2^>80, see S1) than the diet-linked morphological index and the niche index (respectively R^2^ = 65 and 68, see S1). Moreover, some community indices could be especially sensitive to one or a few species with extreme values of originality. In this study, it was the case with the European eel, which disproportionately increased the original community indices based on phylogeny and on habitat-linked morphology. This is the main purpose of this index, to weight unique species. However, because the European eel is classified as critically endangered at the national and global scales by the IUCN, these original indices also met in this particular case, the red list species indicator. Moreover, the European eel is a patrimonial species, there is a strong cultural heritage in France associated with this species for their fishing and cooking and thus for their taste, but also for their unique form and shape. For this last one based on the human vision, an originality index based on morphology could be common avenues for all species to be “objectively” quantified on the arguments develop by naturalists trying to preserve the unique forms and shapes that have been emerging on Earth.

### II. Spatio-temporal patterns

We found that for the river fish communities, mapping each diversity component separately reveals partially congruent patterns between functional or phylogenetic originality, and specialization. All indices highlight Corsica, and to a lesser extent the Channel watersheds, as hotspots of originality and specialization. In contrast, the Seine watershed presents a less original and specialized fish community. For all the other watersheds, the different functional and phylogenetic COI and CSI are not congruent. For example, diet-linked and habitat-linked morphological COI present completely different patterns. The former highlights a strong originality in all small rivers especially mountainous streams, whereas the latter did not present any strong variation pattern at all, except for Corsica Island. 

These common patterns suggest that species occurring locally may be derived from regional species pools with similar biogeographical and evolutionary histories [63,64]. Moreover, for a given regional pool, species may respond to environmental gradients in different ways affecting the spatial distribution of the different biodiversity components and generating a spatial mismatch between functional and phylogenetic COI and CSI [64,65]. These results challenge the use of a single component as a surrogate for the others, and stress the need to first understand the different processes underlying each index and second to adopt a more integrative approach for conservation. One option to deal with the different messages given by the functional properties of communities and the resulting set of measurements is to be able to provide a hierarchy of their meanings depending on the context and perspectives or more reasonable to only use common patterns. 

A temporal effect has been detected for three original community indices (Table 3). Both morphological and Niche community indices significantly increased over time. This temporal dynamic should result from a global increase of the total species abundance, which has been shown (t= 5,09; p<0.001) and thus, may be the result of a global improvement of the entire river ecosystems. Indeed, global water quality has improved compared to the last century thanks to significant efforts to decrease organic substances [26,66]. Moreover, fish populations in Europe are still in their re-colonizing process since the last glaciations and some species are expected to extend their geographical area [67]. More precisely, each index is more influenced by the population dynamic of a few species presenting a high original value. The European eel has a very high original value especially for both habitat-linked morphological and niche indices, even though this species is declining (t= -6,53; p<0.001) which implies an important increase by other species in compensation. For the niche-COI, the global increase could be mainly linked with the population expansion of the European perch, northern pike, and minnow. The northern pike is very popular with anglers favoring their introduction and thus, may have a positive impact on the population dynamic [26]. In the case of the diet-linked morphological-COI, it could be explained by three increasing species: the three spines stickleback, the common nase, and again the minnow. And in the case of the habitat-linked morphological-COI, the temporal increase could be mainly linked with the increase of two introduced species: the crucian carp and the pumpkinseed sunfish. Introduced species may increase faster than native species due to their rapid spread and their repeated introductions (accidental or deliberate). 

### III. Community Indicators

If originality and specialization approaches are completely different in their mathematical calculations, they describe complementary components of the functional properties of communities with similar expectations for their roles in ecosystem services. It has been theoretically and experimentally shown that the alteration of biodiversity disrupts ecological functions performed by species assemblages [68], and we know that species niche partitioning is fundamental in ecosystems properties [69]. Thus the more we are losing specialist or original species, the more we are losing irreplaceable functions in the ecosystem [6,70]. 

The theoretical background underlying the link between COI, CSI, and ecosystem functioning is growing in the literature, but it does not yet mean that both types of indices are relevant as functional biodiversity indicators. Community indicators have to be sensitive to anthropogenic pressures and give a clear and simple message to be technically and practically used by the targeted audience. Thus, the interpretation of indicators has to be as simple as possible and some communication qualities have to be accounting for. An indicator that is well recognized by all biodiversity stakeholders is more likely to be used in the future. According to the Millennium Ecosystem Assessment [17], one of the most important direct drivers of biodiversity loss and ecosystem service changes are land use modifications, including the physical modification of rivers. In this study we build innovative indices for community river fish in order to open the way for a new generation of indicators based on traits or niche linked with ecosystem functions. We compared each of our created community indices with the land use dataset (Figure 6). Interestingly the CSI present a very high score in urban areas. Fish communities are composed of specialist species in this artificialized habitat, probably because the environmental filter is very strong and species need to be specialists of this disturbed area such as the Black bullhead (*Ameiurus melas*), an invasive species which have a strong SSI. This pattern of urban specialist species in fish seems to be similar to the bird one, with urban specialist species such as pigeons (*Colombia liva*) or house sparrows (*Passer domesticus*) [21].

One COI appears particularly relevant to become a functional indicator of river fish communities, the COI based on species niche. This functional COI is sensitive to the different kinds of human disturbances with a simple interpretation: higher disturbance correlates with lower indicator values. Indeed, when we sorted land use on an artificialization gradient from natural to farming to urban areas, we observed a decrease in the niche-COI (Figure 6). Moreover, the three different variations of the CORINE Land cover data set give exactly the same results for the niche-COI, and thus we can be confident in its reproducibility over classification criteria. Finally, we evaluated its sensitivity to spatial scale. Is the pattern observed at the national scale still present at the regional scale? We observed a consistent pattern over the different watersheds, with the exception of the Mediterranean region (Figure 7), which may be due to the sample size. More interestingly, but not useful as an indicator, it may due to a local scale dependence or an eco-regional dependence. Indeed, Mediterranean watershed is very small and without any big river. In addition, all the urban areas are concentrated along the coast in this part of the Mediterranean eco-region. Further investigations need to be done to confirm the context of the use of this potential bio-indicator.

Otherwise, Niche-COI as a functional biodiversity indicator encompasses species indicators like the CBD headline indicator “trend of selected species” because they consider a complete ecological group with their functions and their common dynamics. As a result, they carry more significant ecological information so that expectations and objectives for biodiversity stakeholders can be derived. Originality indices have already been used as indicators in biodiversity conservation contexts. Isaac et al. [71] built one called EDGE (Evolutionary Distinct and Globally Endangered) based on the phylogenetic originality and conservation status of species. At the community level, Mouillot et al. [70] have suggested functional and phylogenetic COI to evaluate conservation action areas. Because interspecific competition is more intense among species sharing common traits due to the limiting similarity principle [72], they expected that under protected areas competition might drive the better colonization or subsistence of the most original species because of niche complementary. We believe that even if one of the goals of this study was to develop functional biodiversity indicators for environmental policy makers, the Niche-COI could also be used at the scale of conservation reserves and may be used by managers of protected areas. 

Finally, we have to keep in mind that biodiversity indicators help to prioritize conservation actions to conserve ecological functions and *in fine* ecosystem “services”. However, evaluation and measure alone are not sufficient in order to stop biodiversity loss, human pressures also must be limited. 

## Supporting Information

File S1 **Complementary analysis: Sensitivity of the SOI to the number of species.** Table S1, List of the studied species.
(DOC)

File S2 **Complete sensitivity analysis results of community indices to human pressures.** Table S2, Complete results of the relation between community indices and land used.
(PDF)

## References

[1] Balmford A, Bennun L, Brink BT, Cooper D, Côté IM et al. (2005) Ecology: The Convention on Biological Diversity's 2010 Target. Science 307: 212–213. doi:10.1126/science.1106281. PubMed: 15653489. 15653489

[2] Mace GM (2005) Biodiversity: an index of intactness. Nature 434: 32–33. doi:10.1038/434032a. PubMed: 15744284. 15744284

[3] Feld CK, Martins da Silva P, Paulo Sousa J, de Bello F, Bugter R et al. (2009) Indicators of biodiversity and ecosystem services: a synthesis across ecosystems and spatial scales. Oikos 118: 1862–1871. doi:10.1111/j.1600-0706.2009.17860.x.

[4] Butchart SHM, Walpole M, Collen B, Van Strien A, Scharlemann JPW et al. (2010) Global biodiversity: indicators of recent declines. Science 328: 1164–1168. doi:10.1126/science.1187512. PubMed: 20430971. 20430971

[5] Mace GM, Norris K, Fitter AH (2012) Biodiversity and ecosystem services: a multilayered relationship. Trends Ecol Evol 27: 19–26. doi:10.1016/j.tree.2011.08.006. PubMed: 21943703. 21943703

[6] Clavel J, Julliard R, Devictor V (2011) Worldwide decline of specialist species: toward a global functional homogenization? Frontiers in Ecology and the Environment 9: 222–228. doi:10.1890/080216.

[7] Cardinale BJ, Duffy JE, Gonzalez A, Hooper DU, Perrings C et al. (2012) Biodiversity loss and its impact on humanity. Nature 486: 59–67. doi:10.1038/nature11148. PubMed: 22678280. 22678280

[8] Tachet H, Bournaud M, Richoux PH (1984) Introduction à l'étude des macroinvertebres des eaux douces. Université Claude Bernard Lyon I, Lyon.

[9] Lenat DR (1988) Water quality assessment of streams using a qualitative collection method for benthic macroinvertebrates. Journal of the North American Benthological Society 7: 222–233. doi:10.2307/1467422.

[10] Oberdorff T, Pont D, Hugueny B, Chessel D (2001) A probabilistic model characterizing fish assemblages of French rivers: a framework for environmental assessment. Freshwater Biol 46: 399–415. doi:10.1046/j.1365-2427.2001.00669.x.

[11] Oberdorff T, Pont D, Hugueny B, Porcher J (2002) Development and validation of a fish-based index for the assessment of “river health” in France. Freshwater Biol 47: 1720–1734. doi:10.1046/j.1365-2427.2002.00884.x.

[12] Baker ME, King RS (2013) Of TITAN and straw men: an appeal for greater understanding of community data. Freshwater. Science 32: 489–506. doi:10.1899/12-142.1.

[13] Berlow EL, Dunne JA, Martinez ND, Stark PB, Williams RJ et al. (2009) Simple prediction of interaction strengths in complex food webs. Proc Natl Acad Sci U S A 106: 187–191. doi:10.1073/pnas.0806823106. PubMed: 19114659. 19114659 PMC2629248

[14] Kéfi S, Berlow EL, Wieters EA, Navarrete SA, Petchey OL et al. (2012) More than a meal… integrating non-feeding interactions into food webs. Ecology Letters 15: 291–300. doi:10.1111/j.1461-0248.2011.01732.x. PubMed: 22313549. 22313549

[15] Devictor V, Julliard R, Clavel J, Jiguet F, Lee A et al. (2008) Functional biotic homogenization of bird communities in disturbed landscapes. Global Ecol Biogeography 17: 252–261. doi:10.1111/j.1466-8238.2007.00364.x.

[16] Millenium. Ecosystem Assessment, synthesis on biodiversity (2005). Available: http://www.millenniumassessment.org/documents/document.354.aspx.pdf.

[17] Baker ME, King RS (2010) A new method for detecting and interpreting biodiversity and ecological community thresholds. Methods in Ecology and Evolution 1: 25–37. doi:10.1111/j.2041-210X.2009.00007.x.

[18] Levrel H (2007) Selecting indicators for the management of biodiversity. Institut francais de la biodiversité, Paris: 1–93.

[19] de Bello F, Lavorel S, Gerhold P, Reier Ü, Pärtel M (2010) A biodiversity monitoring framework for practical conservation of grasslands and shrublands. Biological Conservation 143: 9–17. doi:10.1016/j.biocon.2009.04.022.

[20] Pavoine S, Ollier S, Dufour A (2005) Is the originality of a species measurable? Ecology Letters 8: 579–586. doi:10.1111/j.1461-0248.2005.00752.x.

[21] Julliard R, Clavel J, Devictor V, Jiguet F, Couvet D (2006) Spatial segregation of specialists and generalists in bird communities. Ecol Lett 9: 1237–1244. doi:10.1111/j.1461-0248.2006.00977.x. PubMed: 17040326. 17040326

[22] Kampichler C, van Turnhout CAM, Devictor V, van der Jeugd HP (2012) Large-Scale Changes in Community Composition: Determining Land Use and Climate Change Signals. PLOS ONE 7: e35272. doi:10.1371/journal.pone.0035272.t002. PubMed: 22523579. 22523579 PMC3327650

[23] Dudgeon D, Arthington AH, Gessner MO, Kawabata Z, Knowler DJ et al. (2006) Freshwater biodiversity: importance, threats, status and conservation challenges. Biological Rev 81: 163-182. doi:10.1017/S1464793105006950. PubMed: 16336747. 16336747

[24] Zalewski M, Cowx IG (1990) Factors affecting the efficiency of electric fishing. In Fishing with Electricity. Applications in Freshwater Fisheries Management Cowx IG Lamarque P Oxford: Blackwell Scientific Publications pp. 89–111.

[25] CEN, 2003 Water quality – sampling of fish with electricity. European Standard – EN 14011: 2003 Brussels: European Committee for Standardization.

[26] Poulet N, Beaulaton L, Dembski S (2011) Time trends in fish populations in metropolitan France: insights from national monitoring data. J Fish Biol 79: 1436–1452. doi:10.1111/j.1095-8649.2011.03084.x. PubMed: 22136235. 22136235

[27] Froese R, Pauly D (2000). FishBase 2000: concepts, design and data sources, Los Baños: International Centre for Living Aquatic Resources Management (distributed with four CD-ROMs; see www.fishbase.org for updates)

[28] http://www.fishbase.org.

[29] Buisson L, Grenouillet G (2009) Contrasted impacts of climate change on stream fish assemblages along an environmental gradient. Diversity and Distributions 15: 613–626. doi:10.1111/j.1472-4642.2009.00565.x.

[30] Gatz AJJ (1979) Ecological morphology of freshwater stream fishes. Tulane Studies in Zoology and Botany 21 : 91–124.

[31] Labropoulou M, Markakis G (1998) Morphological-dietary relationships within two assemblages of marine demersal fishes. Environ Biol. Fish 51: 309–319. doi:10.1023/A:1007445112309.

[32] Sibbing FA, Nagelkerke LAJ (2001) Resource partitioning by Lake Tana barbs predicted from fish morphometrics and prey characteristics. Rev Fish Biol Fisheries 10: 383–437.

[33] Kottelat M, Freyhof J (2007) Handbook of European fresh- water fishes. Kottelat, Cornol. Switzerland and Freyhof, Berlin, Germany.

[34] Berg LS (1965) Fishes of the USSR and adjacent countries, Vol. I- III, 4th edn. Israel Program for Scientific Translation, Jerusalem.

[35] Schleuter D, Daufresne M, Veslot J, Mason NWH, Lanoiselée C et al. (2012) Geographic isolation and climate govern the functional diversity of native fish communities in European drainage basins. Global Ecol Biogeography. 21: 1083-1095. doi:10.1111/j.1466-8238.2012.00763.x.

[36] Swofford D (2002) PAUP: Phylogenetic Analysis Using Parsimony. Sinauer, Sunderland, MA.

[37] Posada D, Buckley TR (2004) Model Selection and Model Averaging in Phylogenetics: Advantages of Akaike Information Criterion and Bayesian Approaches Over Likelihood Ratio Tests. Syst Biol 53: 793–808. doi:10.1080/10635150490522304. PubMed: 15545256. 15545256

[38] Grenouillet G, Buisson L, Casajus N, Lek S (2011) Ensemble modelling of species distribution: the effects of geographical and environmental ranges. Ecography 34: 9–17. doi:10.1111/j.1600-0587.2010.06152.x.

[39] Bossard M, Feranec J, Othael J (2000) CORINE Land Cover Technical Guide—Addendum 2000. European Environment Agency website. Technical Report available. Available: http://www.eea.europa.eu/publications/tech40add. Accessed 2013 October 23

[40] Foley JA (2005) Global Consequences of Land Use. Science 309: 570–574. doi:10.1126/science.1111772. PubMed: 16040698. 16040698

[41] Sliva L, Williams DD (2001) Buffer zone versus whole catchment approaches to studying land use impact on river water quality. Water Res 35: 3462–3472. doi:10.1016/S0043-1354(01)00062-8. PubMed: 11547869. 11547869

[42] Wang L, Lyons J, Rasmussen P, Seelbach P, Simon T et al. (2003) Watershed, reach, and riparian influences on stream fish assemblages in the Northern Lakes and Forest Ecoregion, U.S.A. Can J Fish Aquat Sci 60: 491–505. doi:10.1139/f03-043.

[43] Marzin A, Verdonschot PFM, Pont D (2012) The relative influence of catchment, riparian corridor, and reach-scale anthropogenic pressures on fish and macroinvertebrate assemblages in French rivers. Hydrobiologia 704: 375–388. doi:10.1007/s10750-012-1254-2.

[44] Chessel D, Dufour AB, Thioulouse J (2004) The ade4 package-I- One-table methods. R NEWS 4: 5-10.

[45] Pinheiro J, Bates D, DebRoy S, Sarkar D and the R Development Core Team (2012). R development core team (2008) R: a language and environment for statistical computing. R Foundation for Statistical Computing, Vienna, Austria ISBN 3-900051-07-0, URL

[46] Pavoine S, Vallet J, Dufour A-B, Gachet S, Daniel H (2009) On the challenge of treating various types of variables: application for improving the measurement of functional diversity. Oikos 118: 391–402. doi:10.1111/j.1600-0706.2008.16668.x.

[47] Rao CR (1982) Diversity and dissimilarity coefficients: a unified approach. Theoretical Population Biology 21: 24–43. doi:10.1016/0040-5809(82)90004-1.

[48] Redding DW, Mooers AØ (2006) Incorporating Evolutionary Measures into Conservation Prioritization. Conserv Biol 20: 1670–1678. doi:10.1111/j.1523-1739.2006.00555.x. PubMed: 17181802. 17181802

[49] Laird NM, Ware JH (1982) Random-Effects Models for Longitudinal Data. Biometrics, 38(4): 963–974. doi:10.2307/2529876. PubMed: 7168798. 7168798

[50] Lindstrom MJ, Bates DM (1988) Newton—Raphson and EM algorithms for linear mixed-effects models for repeated-measures data. Journal of the American Statistical Association 83: 1014–1022. doi:10.2307/2290128.

[51] Blum MGB, Heyer E, François O, Austerlitz F (2006) Matrilineal Fertility Inheritance Detected in Hunter-Gatherer Populations Using the Imbalance of Gene Genealogies. PLoS Genet 2: e122. doi:10.1371/journal.pgen.0020122. PubMed: 16933997. 16933997 PMC1526766

[52] Böhning-Gaese K, Oberrath R (1999) Phylogenetic effects on morphological, life-history, behavioural and ecological traits of birds. Evolutionary Ecology Research 1: 347–364.

[53] Diaz S, Cabido M (2001) Vive la difference: plant functional diversity matters to ecosystem processes. Trends in Ecology and Evolution 16: 646–655. doi:10.1016/S0169-5347(01)02283-2.

[54] Sax DF, Gaines SD (2003) Species diversity: from global decreases to local increases. Trends in Ecology and Evolution 18: 561–566. doi:10.1016/S0169-5347(03)00224-6.

[55] Rahel FJ, Hubert WA (1991) Fish assemblages and habitat gradients in a Rocky Mountain–Great Plains stream: biotic zonation and additive patterns of community change. Transactions of the American Fisheries Society 120: 319–322. doi:10.1577/1548-8659(1991)120.

[56] Buisson L, Thuiller W, Lek S, Lim P, Grenouillet G (2008) Climate change hastens the turnover of stream fish assemblages. Global Change Biol 14: 2232–2248. doi:10.1111/j.1365-2486.2008.01657.x.

[57] Petchey OL, Gaston KJ (2006) Functional diversity: back to basics and looking forward. Ecol Lett 9: 741–758. doi:10.1111/j.1461-0248.2006.00924.x. PubMed: 16706917. 16706917

[58] Messier J, McGill BJ, Lechowicz MJ (2010) How do traits vary across ecological scales? A case for trait-based ecology. Ecol Lett 13: 838–848. doi:10.1111/j.1461-0248.2010.01476.x. PubMed: 20482582. 20482582

[59] Schleuter D, Daufresne M, Massol F, Argillier C (2010) A user's guide to functional diversity indices. Ecological Monographs 80: 469–484. doi:10.1890/08-2225.1.

[60] Devictor V, Robert A (2009) Measuring community responses to large-scale disturbance in conservation biogeography. Diversity and Distributions 15: 122–130. doi:10.1111/j.1472-4642.2008.00510.x.

[61] Lepš J, de Bello F, Lavorel S, Berman S (2006) Quantifying and interpreting functional diversity of natural communities: practical considerations matter. Preslia 78: 481–501.

[62] MacColl; ADC (2011) The ecological causes of evolution. Trends Ecol Evol 26: 514–522. doi:10.1016/j.tree.2011.06.009. PubMed: 21763030. Available online at: 10.1016/j.tree.2011.06.009 Available online at: PubMed: 21763030 21763030

[63] Losos JB (2008) Phylogenetic niche conservatism, phylogenetic signal and the relationship between phylogenetic relatedness and ecological similarity among species. Ecol Lett 11: 995–1003. doi:10.1111/j.1461-0248.2008.01229.x. PubMed: 18673385. 18673385

[64] Prinzing A, Reiffers R, Braakhekke WG, Hennekens SM, Tackenberg O et al. (2008) Less lineages more trait variation: phylogenetically clustered plant communities are functionally more diverse. Ecol Lett 11: 809–819. doi:10.1111/j.1461-0248.2008.01189.x. PubMed: 18445034. 18445034

[65] Devictor V, Mouillot D, Meynard C, Jiguet F, Thuiller W et al. (2010) Spatial mismatch and congruence between taxonomic, phylogenetic and functional diversity: the need for integrative conservation strategies in a changing world. Ecol Lett 13: 1030-1040. doi:10.1111/j.1461-0248.2010.01493.x. PubMed: 20545736. 20545736

[66] Dubois A (2009) La qualité des rivières s’améliore pour plusieurs polluants, à l’exception des nitrates. Commissariat Général au Développement Durable MEDEM 18: 1–4.

[67] Reyjol Y, Hugueny B, Pont D, Bianco PG, Beier U et al. (2007) Patterns in species richness and endemism of European freshwater fish. Global Ecol Biogeography 16: 65-75. doi:10.1111/j.1466-822X.2006.00264.x.

[68] Hughes TP, Baird AH, Bellwood DR, Card M, Connolly SR et al. (2003) Climate change, human impacts, and the resilience of coral reefs. Science 301: 929–933. doi:10.1126/science.1085046. PubMed: 12920289. 12920289

[69] Loreau M, de Mazancourt C (2008) Species Synchrony and Its Drivers: Neutral and Nonneutral Community Dynamics in Fluctuating. Environments - Am Nat 172: E48–E66. doi:10.1086/589746. 18598188

[70] Mouillot D, Culioli JM, Pelletier D, Tomasini JA (2008) Do we protect biological originality in protected areas? A new index and an application to the Bonifacio Strait Natural Reserve. Biological Conservation 141: 1569–1580. doi:10.1016/j.biocon.2008.04.002.

[71] Isaac NJB, Turvey ST, Collen B, Waterman C, Baillie JEM (2007) Mammals on the EDGE: Conservation Priorities Based on Threat and Phylogeny. PLOS ONE 2: e296. doi:10.1371/journal.pone.0000296.g003. PubMed: 17375184. 17375184 PMC1808424

[72] MacArthur R, Levins R (1967) The limiting similarity, convergence, and divergence of coexisting species. American Naturalist 101: 377–385. doi:10.1086/282505.

