# Supplementary material for: A New Freshwater Biodiversity Indicator Based on Fish Community Assemblages
Source: PLoS One. 2013 Nov 22;8(11):e80968. doi: 10.1371/journal.pone.0080968 (PMC3838364; doi:10.1371/journal.pone.0080968)
Supplement: File S1 — Complementary analysis: Sensitivity of the SOI to the number of species. Table S1, List of the studied species. (DOC) [file pone.0080968.s001.doc]

**Supplementary Materials 1 – S1.**

Complementary analysis: Sensitivity of the SOI to the number of species.

We tested the sensitivity of the Species Originality Index (SOI) based on traits data set to the addition of species in the initial dataset. Because our originality index is based on distance metric the originality specie score may be variable in function of the number of species studied.

Using the same methods as described in the paper we calculated new SOI including a greater number of species in the dataset. The SOI based on the diet and the habitat linked-morphology contained 41 species, which represent more than 96% of the abundance of total catch (Table1 S2). Whereas the SOI based on the life history traits and the niche contained 45 species, which represent around 97% of the abundance of total catch (Table1 S2).

We investigated correlation between each new SOI and the SOI used in the paper based on 26 species. The life history traits index and the habitat-linked morphological index were strongly correlated (respectively R^2^=81, R^2^=86). The diet-linked morphological index and the niche index were less correlated (respectively R^2^ = 65 and 68).

**Table S1.** List of the studied species.

| **Latin species name** | **THV and Niche data** | **Morpho data** |
| --- | --- | --- |
| *Abramis brama* | - | - |
| *Alburnoides bipunctatus* | - | - |
| *Alburnus alburnus* | - | - |
| *Ameiurus melas* | - | - |
| *Anguilla anguilla* | - | - |
| *Barbatula barbatula* | - | - |
| *Barbus barbus* | - | - |
| *Barbus meridionalis* | - | - |
| *Blicca bjoerkna* | - |  |
| *Carassius auratus* | - |  |
| *Carassius carassius* | - | - |
| *Chondrostoma nasus* | - | - |
| *Cottus gobio* | - | - |
| *Cyprinus carpio* | - | - |
| *Esox lucius* | - | - |
| *Gambusia affinis* | - | - |
| *Gasterosteus aculeatus* | - | - |
| *Gobio gobio* | - | - |
| *Gymnocephalus cernuus* | - | - |
| *Hypophthalmichthys molitrix* | - | - |
| *Lampetra fluviatilis* | - |  |
| *Lampetra planeri* | - |  |
| *Lepomis gibbosus* | - | - |
| *Leuciscus leuciscus* | - | - |
| *Lota lota* | - | - |
| *Micropterus salmoides* | - | - |
| *Oncorhynchus mykiss* | - | - |
| *Parachondrostoma toxostoma* | - | - |
| *Perca fluviatilis* | - | - |
| *Phoxinus phoxinus* | - | - |
| *Pseudorasbora parva* | - | - |
| *Pungitius pungitius* | - | - |
| *Rhodeus amarus* | - | - |
| *Rutilus rutilus* | - | - |
| *Salmo salar* | - | - |
| *Salmo trutta* | - | - |
| *Salvelinus fontinalis* | - | - |
| *Sander lucioperca* | - | - |
| *Scardinius erythrophthalmus* | - | - |
| *Silurus glanis* | - | - |
| *Squalius cephalus* | - | - |
| *Telestes souffia* | - | - |
| *Thymallus thymallus* | - | - |
| *Tinca tinca* | - | - |
| *Umbra pygmaea* | - | - |
