# Supplementary material for: A New Freshwater Biodiversity Indicator Based on Fish Community Assemblages
Source: PLoS One. 2013 Nov 22;8(11):e80968. doi: 10.1371/journal.pone.0080968 (PMC3838364; doi:10.1371/journal.pone.0080968)
Supplement: File S2 — Complete sensitivity analysis results of community indices to human pressures. Table S2, Complete results of the relation between community indices and land used. (PDF) [file pone.0080968.s002.pdf]

Supplementary material 2 – S2

Table S2. Complete results of the relation between Community indices and land used.

We tested the relationships between Community indices and landscape variables using mixed-effects linear models with sampling site as a random effect. We took into account the temporal (year) and spatial effect (geographical coordinates or watersheds) with their interactions. Because no R-squared can be calculated with random effect, we only obtained a proxy of the R-squared with the same model without the random effect. We corrected the p-values for multiple tests using the Benjamini Yekutieli False Discovery Rate. We present here the results of the CORINE Land Cover dataset and its two variations: (i) EuroWaterNet (a special variant of CORINE Land Cover for freshwater common to the European scale), (ii) ONEMA land use classification (a special variant of CORINE Land Cover for freshwater common to the national scale). We present here all results for each best model selected on the AIC.

CORINE Land Cover

Index	Value	Int	yr	Rhi	Sei	Cha	Atl	Loi	Gar	Rho	Med	Cor	x	y	Mea	Far	Mix	Urb	yr:Rhi	yr:Sei	yr:Cha	yr:Atl	yr:Loi	yr:Gar	yr:Rho	yr:Med	yr:Cor
**LHT-COI**	Coef	26	-0.013	-25	-27	-22	-25	-27	-24	-26	-24	-18	5.8e-09	4.3e-08	-0.03	-0.014	0.0029	0.03	0.012	0.013	0.011	0.012	0.013	0.012	0.013	0.012	0.0091
	Std.Error	4.7	0.0023	4.7	4.7	4.7	4.7	4.7	4.7	4.7	4.7	4.9	8.2e-09	7.5e-09	0.0026	0.002	0.02	0.008	0.0023	0.0023	0.0023	0.0023	0.0023	0.0023	0.0024	0.0024	0.0025
	DF	8000	8000	5000	5000	5000	5000	5000	5000	5000	5000	5000	5000	5000	5000	5000	5000	5000	8000	8000	8000	8000	8000	8000	8000	8000	8000
	t-value	5.6	-5.6	-5.2	-5.8	-4.8	-5.3	-5.7	-5.2	-5.5	-5.1	-3.7	0.71	5.7	-11	-7	0.15	3.8	5.2	5.8	4.8	5.3	5.7	5.2	5.5	5.1	3.7
	p-value	<0.001	<0.001	<0.001	<0.001	<0.001	<0.001	<0.001	<0.001	<0.001	<0.001	0.0011	1	<0.001	<0.001	<0.001	1	<0.001	<0.001	<0.001	<0.001	<0.001	<0.001	<0.001	<0.001	<0.001	<0.001
**Niche-COI**	Coef	15	-0.0073	-15	-18	-11	-14	-14	-16	-15	-13	-7.1	2.8e-08	1.1e-09	-0.011	-0.02	-0.016	-0.02	0.0072	0.009	0.0054	0.007	0.0072	0.0078	0.0076	0.0063	0.0036
	Std.Error	4.5	0.0023	4.5	4.5	4.5	4.5	4.5	4.5	4.5	4.6	4.7	7.8e-09	7.1e-09	0.0025	0.0019	0.019	0.0076	0.0023	0.0023	0.0023	0.0023	0.0023	0.0023	0.0023	0.0023	0.0024
	DF	8000	8000	5000	5000	5000	5000	5000	5000	5000	5000	5000	5000	5000	5000	5000	5000	5000	8000	8000	8000	8000	8000	8000	8000	8000	8000
	t-value	3.3	-3.2	-3.2	-4	-2.4	-3.1	-3.2	-3.4	-3.4	-2.8	-1.5	3.5	0.16	-4.5	-10	-0.87	-2.7	3.2	4	2.4	3.1	3.2	3.4	3.4	2.8	1.5
	p-value	0.0095	0.0095	0.0095	0.0016	0.078	0.012	0.0095	0.0088	0.0088	0.03	0.56	0.0078	1	<0.001	<0.001	1	0.039	0.0095	0.0016	0.078	0.012	0.0095	0.0088	0.0088	0.03	0.54
**Habitat-COI**	Coef	44	-0.022	-41	-45	-34	-41	-43	-42	-43	-39	-23	3.1e-08	4.4e-08	-0.01	0.0033	0.0017	0.058	0.021	0.022	0.017	0.02	0.022	0.021	0.022	0.019	0.012
	Std.Error	7.6	0.0038	7.7	7.7	7.7	7.7	7.7	7.7	7.7	7.7	8	1.3e-08	1.2e-08	0.0043	0.0033	0.033	0.013	0.0038	0.0038	0.0038	0.0038	0.0038	0.0038	0.0038	0.0038	0.004
	DF	8000	8000	5000	5000	5000	5000	5000	5000	5000	5000	5000	5000	5000	5000	5000	5000	5000	8000	8000	8000	8000	8000	8000	8000	8000	8000
	t-value	5.7	-5.7	-5.4	-5.9	-4.5	-5.3	-5.7	-5.5	-5.6	-5	-2.9	2.3	3.6	-2.3	1	0.053	4.4	5.4	5.9	4.5	5.3	5.7	5.5	5.6	5	2.9
	p-value	<0.001	<0.001	<0.001	<0.001	<0.001	<0.001	<0.001	<0.001	<0.001	<0.001	0.018	0.085	0.0015	0.085	1	1	<0.001	<0.001	<0.001	<0.001	<0.001	<0.001	<0.001	<0.001	<0.001	0.015
**Phylo-COI**	Coef	45	-0.023	-46	-51	-41	-44	-44	-44	-45	-43	-26	9.8e-08	6.8e-08	-0.0097	-0.027	-0.057	-0.1	0.023	0.025	0.02	0.022	0.022	0.022	0.022	0.022	0.013
	Std.Error	11	0.0054	11	11	11	11	11	11	11	11	11	1.9e-08	1.7e-08	0.0062	0.0047	0.047	0.019	0.0054	0.0054	0.0054	0.0054	0.0054	0.0054	0.0054	0.0055	0.0057
	DF	8000	8000	5000	5000	5000	5000	5000	5000	5000	5000	5000	5000	5000	5000	5000	5000	5000	8000	8000	8000	8000	8000	8000	8000	8000	8000
	t-value	4.2	-4.2	-4.3	-4.6	-3.7	-4.1	-4.1	-4	-4.1	-4	-2.3	5.1	3.9	-1.6	-5.7	-1.2	-5.4	4.3	4.7	3.7	4.1	4.1	4	4.1	4	2.3
	p-value	<0.001	<0.001	<0.001	<0.001	<0.001	<0.001	<0.001	<0.001	<0.001	<0.001	0.097	<0.001	<0.001	0.46	<0.001	0.85	<0.001	<0.001	<0.001	<0.001	<0.001	<0.001	<0.001	<0.001	<0.001	0.092

CORINE Land Cover

Index	Value	Int	yr	Rhi	Sei	Cha	Atl	Loi	Gar	Rho	Med	Cor	x	y	Mea	Far	Mix	Urb	yr:Rhi	yr:Sei	yr:Cha	yr:Atl	yr:Loi	yr:Gar	yr:Rho	yr:Med	yr:Cor
**CSI**	Coef	40	-0.02	-34	-37	-31	-37	-41	-33	-39	-33	-17			-0.042	0.00032	0.035	0.15	0.017	0.019	0.015	0.018	0.02	0.017	0.019	0.017	0.0089
	Std.Error	11	0.0053	11	11	11	11	11	11	11	11	11			0.0061	0.0044	0.047	0.019	0.0053	0.0053	0.0053	0.0053	0.0053	0.0053	0.0053	0.0053	0.0055
	DF	8000	8000	5000	5000	5000	5000	5000	5000	5000	5000	5000			5000	5000	5000	5000	8000	8000	8000	8000	8000	8000	8000	8000	8000
	t-value	3.7	-3.7	-3.2	-3.5	-2.9	-3.5	-3.8	-3.1	-3.6	-3.1	-1.6			-6.8	0.073	0.76	7.8	3.2	3.5	2.9	3.5	3.8	3.1	3.6	3.1	1.6
	p-value	0.0032	0.0032	0.0084	0.004	0.017	0.0042	0.003	0.0096	0.0033	0.0096	0.48			<0.001	1	1	<0.001	0.0084	0.004	0.017	0.0042	0.003	0.0096	0.0033	0.0096	0.46

Index	Value	Int	yr	x	y	Mea	Far	Mix	Urb
**Diet-COI**	Coef	0.3	-6.4e-06	-2.0e-08	-2.7e-08	-0.0056	-0.0078	-0.018	-0.033
	Std.Error	0.095	4.7e-05	2.9e-09	2.6e-09	0.0018	0.0014	0.014	0.0056
	DF	8000	8000	5000	5000	5000	5000	5000	5000
	t-value	3.2	-0.14	-6.7	-10	-3.1	-5.6	-1.3	-5.8
	p-value	0.0062	1	<0.001	<0.001	0.0062	<0.001	0.64	<0.001

ONEMA

Index	Value	(Intercept)	yr	Rhi	Sei	Cha	Atl	Loi	Gar	Rho	Med	Cor	x	y	Mea	Far	Int. Far	Mix	Urb	Int. Urb	yr:Rhi	yr:Sei	yr:Cha	yr:Atl	yr:Loi	yr:Gar	yr:Rho	yr:Med	yr:Cor
**LHT-COI**	Coef	27	-0.013	-25	-28	-23	-26	-27	-25	-26	-25	-19	4.7e-09	4.2e-08	-0.027	-0.016	-0.018	0.011	-0.00068	0.0099	0.013	0.014	0.011	0.013	0.014	0.012	0.013	0.012	0.0094
	Std.Error	4.7	0.0023	4.7	4.7	4.7	4.7	4.7	4.7	4.7	4.7	4.9	8.2e-09	7.5e-09	0.0028	0.0025	0.003	0.0038	0.0028	0.004	0.0023	0.0023	0.0023	0.0023	0.0023	0.0023	0.0023	0.0024	0.0024
	DF	8000	8000	5000	5000	5000	5000	5000	5000	5000	5000	5000	5000	5000	5000	5000	5000	5000	5000	5000	8000	8000	8000	8000	8000	8000	8000	8000	8000
	t-value	5.7	-5.7	-5.4	-5.9	-4.9	-5.4	-5.8	-5.3	-5.6	-5.2	-3.8	0.58	5.6	-9.8	-6.2	-5.9	2.8	-0.24	2.5	5.4	5.9	4.9	5.4	5.8	5.3	5.6	5.2	3.8
	p-value	<0.001	<0.001	<0.001	<0.001	<0.001	<0.001	<0.001	<0.001	<0.001	<0.001	<0.001	1	<0.001	<0.001	<0.001	<0.001	0.021	1	0.055	<0.001	<0.001	<0.001	<0.001	<0.001	<0.001	<0.001	<0.001	<0.001
**Niche-COI**	Coef	16	-0.0077	-15	-19	-12	-15	-15	-16	-16	-13	-7.9	2.7e-08	2.2e-09	-0.01	-0.021	-0.024	-0.015	-0.0049	-0.012	0.0076	0.0095	0.0058	0.0074	0.0076	0.0082	0.008	0.0067	0.004
	Std.Error	4.5	0.0023	4.5	4.5	4.5	4.5	4.5	4.5	4.5	4.6	4.7	7.8e-09	7.1e-09	0.0027	0.0024	0.0028	0.0036	0.0027	0.0038	0.0023	0.0023	0.0023	0.0023	0.0023	0.0023	0.0023	0.0023	0.0024
	DF	8000	8000	5000	5000	5000	5000	5000	5000	5000	5000	5000	5000	5000	5000	5000	5000	5000	5000	5000	8000	8000	8000	8000	8000	8000	8000	8000	8000
	t-value	3.4	-3.4	-3.4	-4.2	-2.6	-3.2	-3.4	-3.6	-3.5	-2.9	-1.7	3.5	0.31	-3.8	-8.8	-8.5	-4.1	-1.9	-3.1	3.4	4.2	2.6	3.3	3.4	3.6	3.5	2.9	1.7
	p-value	0.0049	0.0049	0.0049	<0.001	0.049	0.0068	0.0049	0.0044	0.0048	0.017	0.39	0.0049	1	0.0024	<0.001	<0.001	0.0011	0.28	0.011	0.0049	<0.001	0.049	0.0068	0.0049	0.0044	0.0048	0.017	0.37
**Habitat-COI**	Coef	44	-0.022	-41	-45	-34	-40	-43	-42	-43	-39	-23	2.2e-08	4.8e-08	-0.0052	0.0032	-0.014	0.051	0.027	0.052	0.02	0.022	0.017	0.02	0.022	0.021	0.021	0.019	0.012
	Std.Error	7.6	0.0038	7.6	7.6	7.6	7.6	7.6	7.6	7.6	7.6	7.9	1.3e-08	1.2e-08	0.0045	0.004	0.0048	0.0062	0.0045	0.0064	0.0038	0.0038	0.0038	0.0038	0.0038	0.0038	0.0038	0.0038	0.004
	DF	8000	8000	5000	5000	5000	5000	5000	5000	5000	5000	5000	5000	5000	5000	5000	5000	5000	5000	5000	8000	8000	8000	8000	8000	8000	8000	8000	8000
	t-value	5.8	-5.7	-5.4	-5.9	-4.5	-5.3	-5.7	-5.5	-5.6	-5.1	-2.9	1.7	3.9	-1.1	0.8	-2.9	8.3	6	8.1	5.4	5.9	4.5	5.3	5.7	5.5	5.6	5.1	3
	p-value	<0.001	<0.001	<0.001	<0.001	<0.001	<0.001	<0.001	<0.001	<0.001	<0.001	0.017	0.39	<0.001	1	1	0.017	<0.001	<0.001	<0.001	<0.001	<0.001	<0.001	<0.001	<0.001	<0.001	<0.001	<0.001	0.015
**Phylo-COI**	Coef	48	-0.024	-49	-53	-43	-46	-46	-46	-47	-45	-28	1.0e-07	6.5e-08	-0.0088	-0.038	-0.028	-0.0097	-0.016	-0.022	0.024	0.026	0.021	0.023	0.023	0.023	0.024	0.023	0.014
	Std.Error	11	0.0054	11	11	11	11	11	11	11	11	11	1.9e-08	1.8e-08	0.0066	0.0059	0.007	0.009	0.0066	0.0094	0.0054	0.0054	0.0054	0.0054	0.0054	0.0054	0.0054	0.0055	0.0057
	DF	8000	8000	5000	5000	5000	5000	5000	5000	5000	5000	5000	5000	5000	5000	5000	5000	5000	5000	5000	8000	8000	8000	8000	8000	8000	8000	8000	8000
	t-value	4.4	-4.4	-4.5	-4.9	-3.9	-4.3	-4.3	-4.2	-4.3	-4.2	-2.5	5.2	3.7	-1.3	-6.5	-4	-1.1	-2.4	-2.4	4.5	4.9	3.9	4.3	4.3	4.2	4.3	4.2	2.5
	p-value	<0.001	<0.001	<0.001	<0.001	<0.001	<0.001	<0.001	<0.001	<0.001	<0.001	0.064	<0.001	0.0013	0.74	<0.001	<0.001	1	0.02	0.077	<0.001	<0.001	<0.001	<0.001	<0.001	<0.001	<0.001	<0.001	0.06

ONEMA

Index	Value	(Intercept)	yr	Rhi	Sei	Cha	Atl	Loi	Gar	Rho	Med	Cor	x	y	Mea	Far	Int. Far	Mix	Urb	Int. Urb	yr:Rhi	yr:Sei	yr:Cha	yr:Atl	yr:Loi	yr:Gar	yr:Rho	yr:Med	yr:Cor
**CSI**	Coef	40	-0.02	-34	-38	-31	-37	-41	-33	-38	-33	-17			-0.033	0.00073	-0.0056	0.075	0.022	0.072	0.017	0.019	0.015	0.018	0.02	0.017	0.019	0.017	0.009
	Std.Error	11	0.0053	11	11	11	11	11	11	11	11	11			0.0065	0.0056	0.0066	0.0087	0.0065	0.0093	0.0053	0.0053	0.0053	0.0053	0.0053	0.0053	0.0053	0.0053	0.0055
	DF	8000	8000	5000	5000	5000	5000	5000	5000	5000	5000	5000			5000	5000	5000	5000	5000	5000	8000	8000	8000	8000	8000	8000	8000	8000	8000
	t-value	3.8	-3.7	-3.2	-3.6	-2.9	-3.5	-3.8	-3.2	-3.6	-3.1	-1.6			-5.1	0.13	-0.84	8.7	3.5	7.8	3.2	3.6	2.9	3.5	3.8	3.1	3.6	3.1	1.6
	p-value	0.0027	0.0028	0.0079	0.0034	0.017	0.004	0.0024	0.0091	0.0032	0.0091	0.48			<0.001	1	1	<0.001	0.004	<0.001	0.0079	0.0034	0.017	0.004	0.0024	0.0091	0.0032	0.0091	0.45

Index	Value	(Intercept)	yr	x	y	Mea	Far	Int. Far	Mix	Urb	Int. Urb
**Diet-COI**	Coef	0.3	-6,00E-06	-1.5e-08	-2.6e-08	-0.0077	-0.011	-0.0069	-0.0084	-0.015	-0.015
	Std.Error	0.095	4.7e-05	3.1e-09	2.6e-09	0.0019	0.0018	0.0021	0.0027	0.0019	0.0028
	DF	8000	8000	5000	5000	5000	5000	5000	5000	5000	5000
	t-value	3.1	-0.13	-4.9	-10	-4	-6.4	-3.3	-3.1	-7.9	-5.2
	p-value	0.0059	1	<0.001	<0.001	<0.001	<0.001	0.0035	0.0059	<0.001	<0.001

EUROWATER

Index	Value	Int	yr	Rhi	Sei	Cha	Atl	Loi	Gar	Rho	Med	Cor	x	y	Mea	Far	Mix	Urb	Int. Urb	yr:Rhi	yr:Sei	yr:Cha	yr:Atl	yr:Loi	yr:Gar	yr:Rho	yr:Med	yr:Cor
**LHT-COI**	Coef	27	-0.013	-25	-28	-23	-25	-27	-25	-26	-24	-18	8.0e-09	3.7e-08	-0.0084	-0.012	0.016	0.002	0.013	0.012	0.014	0.011	0.013	0.014	0.012	0.013	0.012	0.0093
	Std.Error	4.7	0.0024	4.7	4.7	4.7	4.7	4.7	4.7	4.7	4.8	4.9	8.2e-09	7.8e-09	0.0025	0.0033	0.0042	0.0032	0.0043	0.0024	0.0024	0.0024	0.0024	0.0024	0.0024	0.0024	0.0024	0.0025
	DF	8000	8000	5000	5000	5000	5000	5000	5000	5000	5000	5000	5000	5000	5000	5000	5000	5000	5000	8000	8000	8000	8000	8000	8000	8000	8000	8000
	t-value	5.6	-5.6	-5.3	-5.8	-4.8	-5.4	-5.8	-5.2	-5.5	-5.1	-3.7	0.98	4.8	-3.4	-3.5	3.8	0.62	3	5.3	5.8	4.9	5.4	5.8	5.2	5.5	5.2	3.8
	p-value	<0.001	<0.001	<0.001	<0.001	<0.001	<0.001	<0.001	<0.001	<0.001	<0.001	0.001	1	<0.001	0.0026	0.0018	<0.001	1	0.01	<0.001	<0.001	<0.001	<0.001	<0.001	<0.001	<0.001	<0.001	<0.001
**Niche-COI**	Coef	16	-0.0078	-16	-19	-12	-15	-16	-17	-16	-14	-8.1	3.6e-08	3.8e-09	-0.012	-0.023	-0.014	-0.0067	-0.013	0.0078	0.0096	0.006	0.0075	0.0078	0.0083	0.0081	0.0069	0.0041
	Std.Error	4.5	0.0023	4.5	4.5	4.5	4.5	4.5	4.5	4.6	4.6	4.8	7.7e-09	7.4e-09	0.0023	0.0031	0.0039	0.003	0.0041	0.0023	0.0023	0.0023	0.0023	0.0023	0.0023	0.0023	0.0023	0.0024
	DF	8000	8000	5000	5000	5000	5000	5000	5000	5000	5000	5000	5000	5000	5000	5000	5000	5000	5000	8000	8000	8000	8000	8000	8000	8000	8000	8000
	t-value	3.5	-3.5	-3.4	-4.2	-2.6	-3.3	-3.4	-3.7	-3.6	-3	-1.7	4.6	0.51	-5.2	-7.3	-3.7	-2.2	-3.2	3.4	4.2	2.6	3.3	3.4	3.7	3.6	3	1.7
	p-value	0.004	0.004	0.004	<0.001	0.04	0.0055	0.004	0.0034	0.0037	0.013	0.36	<0.001	1	<0.001	<0.001	0.0034	0.03	0.0079	0.004	<0.001	0.039	0.0055	0.004	0.0034	0.0037	0.013	0.34
**Habitat-COI**	Coef	43	-0.022	-41	-45	-34	-40	-43	-42	-43	-38	-23	1.9e-08	4.8e-08	-9e-04	-0.015	0.05	0.027	0.051	0.02	0.022	0.017	0.02	0.022	0.021	0.021	0.019	0.012
	Std.Error	7.6	0.0038	7.6	7.6	7.6	7.6	7.6	7.6	7.6	7.6	7.9	1.3e-08	1.2e-08	0.0039	0.0053	0.0067	0.0052	0.0069	0.0038	0.0038	0.0038	0.0038	0.0038	0.0038	0.0038	0.0038	0.004
	DF	8000	8000	5000	5000	5000	5000	5000	5000	5000	5000	5000	5000	5000	5000	5000	5000	5000	5000	8000	8000	8000	8000	8000	8000	8000	8000	8000
	t-value	5.7	-5.7	-5.4	-5.9	-4.5	-5.3	-5.7	-5.5	-5.6	-5	-2.9	1.4	3.9	-0.23	-2.8	7.6	5.2	7.4	5.4	5.9	4.5	5.3	5.7	5.5	5.6	5	2.9
	p-value	<0.001	<0.001	<0.001	<0.001	<0.001	<0.001	<0.001	<0.001	<0.001	<0.001	0.018	0.6	<0.001	1	0.022	<0.001	<0.001	<0.001	<0.001	<0.001	<0.001	<0.001	<0.001	<0.001	<0.001	<0.001	0.015
**Phylo-COI**	Coef	48	-0.024	-49	-54	-43	-47	-47	-47	-48	-46	-29	1.2e-07	7.3e-08	-0.025	-0.031	-0.014	-0.023	-0.029	0.025	0.027	0.022	0.024	0.023	0.023	0.024	0.023	0.014
	Std.Error	11	0.0054	11	11	11	11	11	11	11	11	11	1.9e-08	1.8e-08	0.0057	0.0077	0.0097	0.0075	0.01	0.0054	0.0054	0.0054	0.0054	0.0054	0.0054	0.0054	0.0055	0.0057
	DF	8000	8000	5000	5000	5000	5000	5000	5000	5000	5000	5000	5000	5000	5000	5000	5000	5000	5000	8000	8000	8000	8000	8000	8000	8000	8000	8000
	t-value	4.5	-4.4	-4.5	-4.9	-4	-4.3	-4.3	-4.3	-4.4	-4.2	-2.5	6.1	4	-4.4	-4	-1.5	-3.1	-2.9	4.5	4.9	4	4.3	4.3	4.3	4.4	4.2	2.5
	p-value	<0.001	<0.001	<0.001	<0.001	<0.001	<0.001	<0.001	<0.001	<0.001	<0.001	0.049	<0.001	<0.001	<0.001	<0.001	0.56	0.0091	0.019	<0.001	<0.001	<0.001	<0.001	<0.001	<0.001	<0.001	<0.001	0.046

Index	Value	Int	yr	Rhi	Sei	Cha	Atl	Loi	Gar	Rho	Med	Cor	Mea	Far	Mix	Urb	Int. Urb	yr:Rhi	yr:Sei	yr:Cha	yr:Atl	yr:Loi	yr:Gar	yr:Rho	yr:Med	yr:Cor
**CSI**	Coef	39	-0.019	-34	-37	-30	-36	-40	-33	-38	-33	-17	0.011	0.01	0.091	0.037	0.086	0.017	0.019	0.015	0.018	0.02	0.016	0.019	0.016	0.0087
	Std.Error	11	0.0053	11	11	11	11	11	11	11	11	11	0.0054	0.0072	0.0093	0.0074	0.0099	0.0053	0.0053	0.0053	0.0053	0.0053	0.0053	0.0053	0.0053	0.0055
	DF	8000	8000	5000	5000	5000	5000	5000	5000	5000	5000	5000	5000	5000	5000	5000	5000	8000	8000	8000	8000	8000	8000	8000	8000	8000
	t-value	3.7	-3.7	-3.2	-3.5	-2.8	-3.4	-3.8	-3.1	-3.6	-3.1	-1.5	2.1	1.4	9.8	5	8.7	3.2	3.5	2.9	3.4	3.8	3.1	3.5	3.1	1.6
	p-value	0.0033	0.0033	0.0097	0.004	0.02	0.0047	0.0029	0.011	0.004	0.011	0.5	0.16	0.63	<0.001	<0.001	<0.001	0.0097	0.004	0.02	0.0047	0.0029	0.011	0.004	0.011	0.47

Index	Value	Int	yr	x	y	Mea	Far	Mix	Urban	Int. Urb
**Diet-COI**	Coef	0.3	-9.4e-06	-1.1e-08	-2.4e-08	-0.011	-0.0094	-0.011	-0.019	-0.018
	Std.Error	0.095	4.7e-05	2.9e-09	2.7e-09	0.0017	0.0023	0.0029	0.0022	0.003
	DF	8000	8000	5000	5000	5000	5000	5000	5000	5000
	t-value	3.2	-0.2	-3.8	-9	-6.5	-4.1	-4	-8.6	-6
	p-value	0.0047	1	<0.001	<0.001	<0.001	<0.001	<0.001	<0.001	<0.001
